# Combined Cavitation and Plasma in Water and Wastewater Remediation—A Review

**DOI:** 10.1021/acsomega.5c08760

**Published:** 2026-01-08

**Authors:** Pengyun Liu, Subramaniam Chidambaranathapillai, Zhilin Wu, Giancarlo Cravotto

**Affiliations:** † Department of Drug Science and Technology, 9314 University of Turin, via P. Giuria 9, Turin 10125, Italy; ‡ College of Chemistry and Chemical Engineering, Key (Guangdong-Hong Kong Joint) Laboratory for Preparation and Application of Ordered Structural Materials of Guangdong Province, 12386 Shantou University, Shantou 515063, China; § Chemistry and Chemical Engineering Guangdong Laboratory, Shantou 515041, China

## Abstract

As novel advanced oxidation processes, combined hydrodynamic cavitation (HC)/plasma and ultrasonic cavitation (UC)/plasma provide distinctive benefits for wastewater treatments, such as being minimal or free of additional additives, relevant fast processes with satisfactory outcomes, and maintaining biocell integrity while avoiding cytotoxin release, prolonged oxidation. A comprehensive review of hybrid processes is essential to recognizing the recent advances in this field, to improving awareness of the related mechanisms, and to guiding future work. Herein, this review presents the mechanisms, synergistic effects, the application, and the impact of critical factors of cavitation/plasma. Furthermore, environmental impacts are stated, and future perspectives are also recommended. Synergism occurred in both degradation and reactive species generation, indicating that cavitation/plasma systems represent one of the most effective hybrid technologies currently available for environmental water remediation.

## Graphical abstract

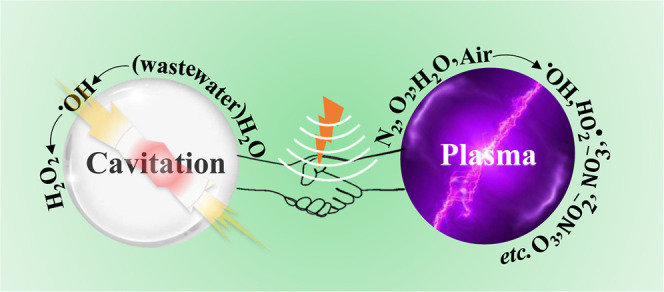

## 1 Introduction

Clean water is essential for life. The expanded world population (up to 10 billion by 2050) and industrialization pose serious challenges to global water scarcity.^1–3^ Wastewater from various industries generally contains complex components and may with toxic and persistent substances, posing potentially harmful effects on humans and aquatic organisms.^4–6^ Moreover, cyanobacterial blooms frequently occur in surface waters due to the increased eutrophication, becoming a major toxicological, hygienic, and technological issue.^7^ Therefore, appropriate wastewater remediation is of great importance to mitigate and prevent the potential environmental risks.^2,4,8–10^ Most of the conventional methods and advanced oxidation processes (AOPs) for wastewater treatment are summarized in Supporting Information Table S1, focusing on their limitations and merits. Nevertheless, global warming, the growing volume of polluted water, and strict water-use and environmental regulations require innovative, safe, robust, efficient, and sustainable treatment approaches.^3,11,12^

Plasma, an overall, electrically neutral gas composed of free electrons, ions, atoms, and molecules, belongs to the fourth state of matter (the other states of matter are solid, liquid, and gas) with properties of high temperature and energetic molecules.^3,13–15^ Plasma can be formed by exerting sufficient energy, in the form of electric or electromagnetic fields, on a liquid (e.g., HC/subatmospheric-pressure plasma (SupCaviPlasma) or gas (e.g., Ar, He, N_2_, O_2_, air, or their mixture). Gas and vapor can be fully or partly ionized, where molecules of the gas dissociate to gas atoms, and move freely to electrons, positive ions, and reactive species.^15,16^ Energy sources for producing plasma include US wave, microwave, laser, alternating current, pulse, and direct current (e.g., direct current-pulse and direct current-direct current).^15,17,18^ The flow of currents through electrodes includes plasma current and ion current, which are derived from plasma production and ion flow in water, respectively. The peak value and number of current pulses decrease with increasing solution conductivities.^5^ The types of plasma production include plasma needle (or jet and torch), streamer, gliding arc discharge, surface plasma discharge (PD), direct current glow discharge, microwave discharge, pulsed corona discharge, dielectric barrier discharge (DBD), pulsed spark discharge, among others.^8,15,19^ Owing to the high temperature and energy densities required, plasmas produce species that are more energetic than ordinary chemicals. These energetic species trigger chemical reactions, even at low temperatures, that are difficult to achieve via more traditional means, forming excited ions, atoms, and molecules rather than selective chemical conversion.^20,21^ Specifically, plasma, in air, is composed of molecules or atoms in their excited or ground states, charged particles (e.g., ions (O^+^, O_2_^+^, and O_2_^–^) and free electrons (e^–^)), neutral species, reactive oxygen and nitrogen species (RONS, including short-life radicals (^•^OH, ^•^O, NO_2_^•^, NO^•^, etc.), metastables (^1^O_2_^•^), and more durable species (O_3_, H_2_O_2_, NO_3_^–^, NO, etc.), UV and vacuum-UV photons. The plasma glow contains excited atoms, free radicals, molecules, and UV radiation.^8,10,22,23^ It has been reported that fewer reactive species are produced in the air than in other gases (e.g., Ar and O_2_) during plasma processes.^24–27^ Unlike electromagnetic discharge-based plasma systems, electrical discharge (ED)-based plasma systems have been widely used in the reported work.^15^ Generally, ED-based plasma systems consist of two electrodes connected to a power source and a working gas, usually requiring a much higher voltage to break down water matrices between electrodes than air.^12^ The electric field improves the generation of vapor microchannels inside electrode gaps.^28^ However, the generated radicals in ED reactors display low mass transfer, limiting the removal efficiency (RE).^28^

Cavitational techniques, another emerging AOP, have also been developed for degrading various pollutants in wastewater.^29^ Cavitation can generally be described as the generation, growth, and collapse of gas- or vapor-filled bubbles (also called cavitation bubbles) in the micro- to nanoscale range, during the rapid pressure change in fluids. The alternation of pressure and flow in fluids can be triggered by geometric constrictions or throttle valves, e.g., orifice plates, venturi, nozzles, and vortex diode.^30–32^ Cavitation bubbles are formed when the local pressure in liquids is below the saturated vapor pressure. The bubbles will continue to expand until they collapse, when they burst, creating high-pressure regions. The abrupt implosion of bubbles leads to the transfer of the internal gas, producing massive point source energy to a tiny spatiotemporal region (known as “hot spots”).^3,9,29,33,34^ Cavitation technologies thus work based on cavitation effects, i.e., mechanical effects (e.g., turbulence, shock waves, high-speed microjets, pressure gradients, counter-jets, shear forces, or whirlpool of liquid), physical effects (e.g., high local pressures and temperatures (up to 1000 atm and 6000 K), luminescence, noise, and erosion), and chemical effects (e.g., the cleavage of H_2_O molecules and the generation of ^•^OH, ^•^OOH, H_2_O_2_, etc.). Cavitation effects accelerate the active species to migrate from gases to liquids, creating extreme physical conditions and abundant ROS in liquids. Microjets and shock waves can cause interparticle collisions at high velocity, these effects can even melt most metals.^2,35,36^ By combining the above-mentioned effects, contaminants can thus be degraded in wastewater. Cavitation can be triggered by the propagation of US (i.e., UC), variations in hydrodynamic flow and pressure (i.e., HC), the rupture of light photons with high intensity (optic cavitation), and other particles (particle cavitation) in liquids. Based on the formation and growth of bubbles, cavitation can be classified as HC, vaporous cavitation, gaseous cavitation, and vibrational cavitation (or UC).^3,9,19,37–42^ Regardless of cavitation type, cavitation bubbles often collapse violently, leading to the relevant cavitation effects.^13^ Pandit, Gogate, and Boczkaj groups have reviewed the HC and UC processes in water and wastewater remediation in detail.^43–45^

The pros and cons of cavitation–plasma combinations for water and wastewater remediation compared to conventional strategies (e.g., ozonation, Fenton reactions, biological oxidation, and UV irradiation) are compiled in Figure 1. Combined HC/plasma and UC/plasma have been used for wastewater remediation in many cases, whereas there is a lack of systematic reviews, especially the deconstruction of their combined mechanisms.^15^ This review focuses on exploring the synergism between HC or UC and plasma as well as presenting the current advances in hybrid wastewater-remediation techniques, and will explore general parameters, the interaction of cavitation and plasma, typical applications, energy efficiency, the effects of critical factors, environmental impacts, and future perspectives.

**Figure 1:**
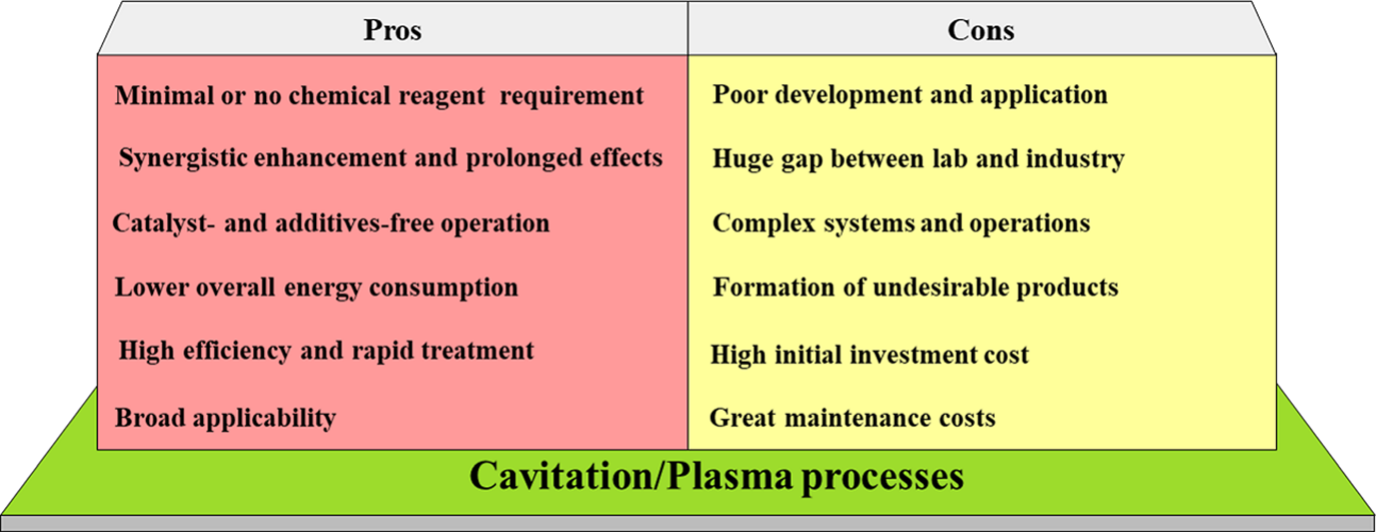
*pros* and *cons* of cavitation–plasma combinations for water and wastewater remediation compared to conventional strategies.

## 2 Mechanisms of Cavitation/Plasma Processes

### 2.1 Mechanism of Plasma

The mechanisms of plasma generation can be clarified via the characteristics of the produced reactive oxidative species (ROS) or RONS.^15^ There are many reactions involved in plasma systems that relate to producing free radicals, ions, and high-energy species (Reactions 1–9).^10,12^ RONS accounts for a large proportion of generated radicals and displays a range of different lifetimes. H_2_O_2_, NO_2_^–^, and NO_3_^–^ possess exceptional transmembrane abilities and can serve as precursors of ROS.^46^ Plasma can also generate ultraviolet (UV) and glow, and trigger cavitation.^3^ Moreover, the collision of atoms in plasma systems is crucial for contaminant degradation since high-energy atoms are developed between the two electrodes from gas phase environments, converting electrical energy to thermal energy under a glow PD.^47–50^

H2O+e−→H+OH•+e− 1

2 H2O+e−→OH•+H++2e−

3 H2O+e−→H•+O•+H•+e−

4 OH•+OH•→H2O2

5 H2O2+UV→OH•+OH•

$$N_{2}+e^{-}\to N_{2}^{+}+{\mathrm{2e}}^{-}$$ 6

7 O2+e−→O•+O•+e−

8 $$O_{2}+O\to O_{3}$$

9 O2+e−→O−+O•

Mechanisms of plasma generation also depend on the ED method. The paths of the plasma-generated species into liquids are of significant importance in contaminant degradation, as plasma can only be ignited in gases in different plasma systems. The properties pros and cons of various plasma processes are listed in Supporting Information Table S2.

Plasma treatments can be performed in both bath and flow-through modes (e.g., sono-plasma).^8^ Underwater PD (or liquid-phase discharge plasma) can take place via spark and streamer mechanisms (Figure 2).^35^ The direct use of ED in a fluid requires sustaining stable PD with high frequencies and voltage pulses, which is quite energy inefficient and restricted by reaction volume and large breakdown voltages (over 10 MV/cm) in water, which is why plasma treatment in volumes over 1 mL has rarely been tested. Although a challenge, introducing the gaseous phase into bulk liquids or on a water surface reduces the breakdown voltage of the medium (1–10 kV/cm), and is very favorable for generating and propagating discharge throughout a liquid. The type of selected gas and their means of injection correlate to the generated RONS and their diffusivity in water. For example, the bubbling of plasma species into liquids can only be applied when air (or N_2_ included mixtures) is used as the discharge gas.^3,8,10,13,51,52^

**Figure 2:**
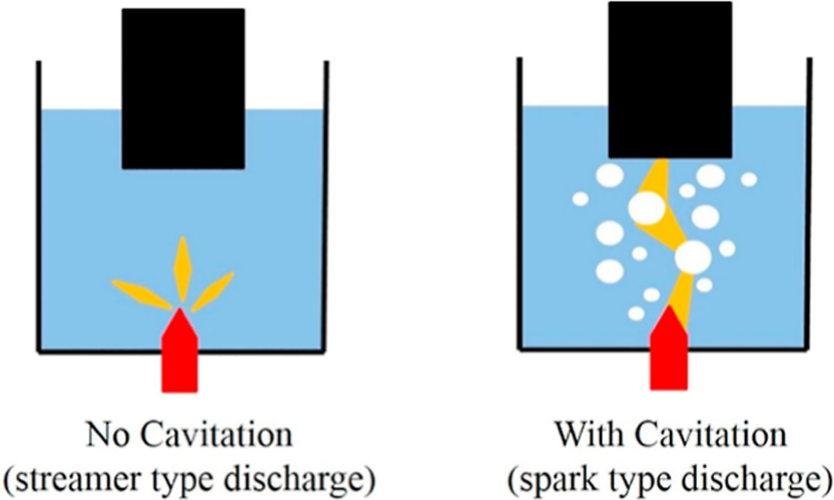
Mechanism of PD with and without UC. Reprinted from ref 35 Copyright (2019), with permission from Elsevier.

As listed in Supporting Information Table S2, plasma processes are environment-friendly, active, free of chemicals and catalysts, rapid, and effective.^15,19^ Owing to the merits mentioned above, plasma has been used as an emerging AOP for wastewater remediation by combining photolysis by UV, high-energy electron radiation, oxidation of ROS or RONS with organic contaminants, microorganism particles, and bacteria.^5,6,29^ Specifically, the antibacterial and bactericidal features of water treated with plasma are mainly due to the formation of H_2_O_2_, ^1^O_2_ and other ROS in bulk liquids.^10^ Oxidation by ^•^OH radicals derived from H_2_O and O_3_ is reported, in most cases, as the main degradation mechanism, followed by direct ozonation and nitrification by N-species.^8^ Plasma-based AOPs can be optimized through controlling operation parameters like treatment time, discharge power, water flow rate, and so forth.^10^ In addition, conductivity is crucial because it depends on treatment substrates and pollution levels, considerably affecting the power consumption of reactors and the plasma production. The conductivity is ∼0.5–3.0 μS/cm for distilled water, ∼4–5 S/m for seawater with a 3% salt concentration, 10^2^ to 10^3^ μS/cm for municipal wastewater and sewage, up to several 10,000 μS/cm for industrial wastewater, and several tens to several thousands of μS/cm for river water.^5^ It is a challenge to introduce gaseous plasma and the relevant reactive species into liquids, and the solutions include igniting the plasma in bubbles (e.g., atmospheric pressure plasma jet) and combining plasma with another method that creates and maintains low-pressure gas conditions inside liquid water (e.g., HC and UC).^13^ When PD involves a single bubble submerged in water, the most intense electric field is located near the bubble interface, thus improving electrical discharge generation.^53,54^ When plasma is generated in air or vapor (water), its treatment performance is determined by the transfer kinetics of radicals from the gas to liquids.^5,6,29^ Plasmas, in combination with AOP and assisted by HC and UC, are probably the most studied approaches at present.

### 2.2 Mechanism of Cavitation

HC releases huge amounts of energy in fluids during the collapse of cavities.^3^ Radical ^•^OH in HC is mainly produced by the dissociation, ionization, and other reactions of gaseous water molecules.^19^ Cavitation number (*C*_v_) and other hydrodynamic factors, including inlet pressure (*P*_i_), flow rate, velocities in the constrictions, the ratio between the total hole perimeter and the total area of the openings, and the ratio between the total hole area and the cross-sectional area of the pipe, etc., are crucial to HC processes.^2,9^ In terms of flow dynamics, HC can typically evolve from incipient cavitation to sheet (attached), followed by developed (cloud-shedding of microbubbles), and super cavitation. Supercavitation is the stage at which the vapor occupies a large volume of fluid and generates a single stable cavity of a large size. The generated cavity usually extends outside the trailing edge of the hydrofoil or other constrictions once the static pressure suddenly reduces in a greater spatiotemporal region.^8,13^ Currently, Venturi throat-, orifice plate-, swirling jet-, vortex diode-, (vortex) rotating-, and self-excited oscillation cavity (pulsed jet)-based HC reactors have been developed, which can be classified as static and dynamic devices.^8^ The static Venturi throat- and orifice plate-based reactors are the most widely used.^9,29^ Owing to the advantages of process acceleration, intensified mass transfer, low consumption of chemicals, scalability, simplicity, easy operations, high energy efficiency, cost-friendly, and safety, etc., HC has been widely used for water treatment. However, HC has the limitations of low energy efficiency and degradation kinetics, and is thus often used as a hybrid technique together with other AOPs and external oxidants (H_2_O_2_, Fenton reagent, O_3_, etc.) to treat antibiotics, dye, etc., in water.^2,3,9,12,55–57^

US can be divided into diagnostic US (1–10 MHz), high-frequency US (0.1–1.0 MHz and 0.1–1.0 W/cm^2^), and low-frequency power US (20–100 kHz and 10–1000 W/cm^2^). The mechanisms of UC effects differ from those in HC. US can trigger two phenomena in liquids, i.e., UC and acoustic streaming.^58^ The propagation of US through a liquid can induce bubble generation and powerful UC to create hot spots, resulting in the pyrolysis of water to form ^•^OH. The generated bubbles will grow in successive compressed and extended cycles, reaching an unstable size of up to ∼150 μm in ∼400 μs. Power US can cause the mechanical effects of cavitation in liquids, altering the physicochemical properties of substances.^14,22^ UC-bubble implosion can ionize H_2_O molecules to generate plasma. At low US intensities, sonoluminescence takes place due to the emission of excited ^•^OH*.^6^ Acoustic streaming (i.e., a macro steady fluid streaming) relates to the reduction of US energy in UC regions. The velocity of acoustic streaming can be as high as 0.4 m/s, reliant on the vibration amplitude, which can greatly improve the mass transfer inside the cavitational reactor.^2,6,35,58^ However, the UC zone is restricted in size due to the significant attenuation of US wave energy that occurs with the distance traveled from the US source to the cavitation zones.^1^ When a liquid is exposed to an intense US field above the cavitation threshold, electric discharge, which features a volumetric glow, can also occur across the entire gap between the electrodes, and this can degrade organic contaminants and microorganisms in water in the absence of additional chemicals.^2,28^ UC is also an AOP and has been used for wastewater remediation, the degradation of pollutants, catalytic reactions, the activation of reagents (e.g., peroxymonosulfate), and the enhancement of other types of AOPs due to the advantages of environmental sustainability, mild treatment conditions, high energy efficiency, simple system design, safety and easy operation, etc.^2,35,46^ UC appears in immobile liquids, whereas HC develops in fluids and is intensified by the channel configuration.^59^ Unlike HC, UC is only generated close to the US radiators, which have limited size due to it generally being designed following the resonance principle, limiting its application in large reaction volumes.^51^ Although organic-contaminant decomposition by sonolysis is poorly cost-effective, insufficient, limited to batch treatment, poor scalability, low reaction volumes, and low mass transfer (as high US frequencies are required), the combination of US with other ROS formation methods or external chemical oxidants is quite a promising approach.^6,12,35^ Our previous works have discussed UC and HC in detail by clarifying their mechanisms, applications, synergies, etc.^60–120^

### 2.3 Mechanism of HC/Plasma

Cavitation techniques can be performed as hybrid processes with plasma processes in three ways: (i) simultaneous cavitation and plasma; (ii) cavitation and plasma in sequence; and (iii) plasma and cavitation in sequence. Most reports involve the first case for water and wastewater remediation, and thus HC/plasma, UC/plasma, and cavitation/plasma refer to the simultaneous processes hereafter if not specifically stated. Generally, HC/plasma processes rely on forming water vapor in fluids downstream of the reactor restrictions, with plasma being generated using high voltage with an electrode pair placed in the bubbles or bubble clouds.^19,63^ The case below presents a detailed electrode system and locations of the cavitation field and electrodes (Figure 3).^7^

**Figure 3:**
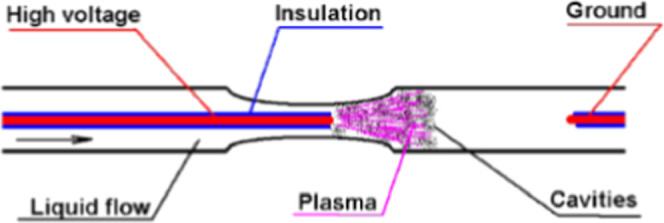
Detailed electrode system and locations of the HC field and electrodes. Reprinted from ref 7 Copyright (2019), with permission from MDPI.

In HC/plasma processes, the size of cavitation bubbles significantly increases during the growth stage, probably with very low gas pressure inside the bubble, which allows for creating microchannels between the electrodes based on Pashen’s law. In simultaneous HC and PD treatment, O_3_ is produced under UV photolysis and the discharges inside HC bubbles. Powerful ^•^OH (2.7 eV) can be generated both at the liquid/gas interface and within bubbles from H_2_O and O_3_ (Reaction 10), along with shockwaves under HC effects. Abundant reactive oxidative species (ROS) can be further produced under PD. Moreover, HC also improves the transfer of reactive species in fluids in HC/plasma systems.^2^ Thus, contaminants in wastewater can be degraded by the above combined effects through Reactions 11–21.^29^ HC has been combined with several plasma processes with differing outcomes. For example, the combination of cavitation bubbles and highly reactive conditions during glow PD (GPD) produced synergistic effects for removing organic pollutants. Due to the low electron mobility in bulk solutions, the charges from the GPD were conducted through the surface of cavitation bubbles. The gas inside HC bubbles can be ionized since this conduction of charges, inducing the generation of more ROS, O_3_ generation, and the UV (300 nm) photolysis, followed by more efficient degradation of contaminants in HC/GPD systems.^64^ HC was combined with cold plasma, where ED can be efficiently propagated between HC bubbles, allowing plasma formation both in the vapor phase and at the gas-bulk liquid interface, as well as the consequent generation of oxidizing (^•^OH, ^•^O, and O_3_) and reducing species (^•^H), thus improving overall mass and energy transfer.^12^ CaviPlasma (i.e., alternating current discharge in a dense HC cloud in liquid) couples efficient mixing of agents and mechanical stress by supercavitation with electro-mechanical stress and UV photolysis, favoring the ignition and maintenance of stable plasmas.^13^ Venturi tube-based HC has been simultaneously used with nanosecond pulse discharge plasma with the help of O_2_ injection (HCAOP), in which the oxidation activation and the generation and utilization of ^•^OH can be improved due to the rapid mass transfer, high internal pressure, large specific surface area, as well as long retention time of microbubbles in liquid. In HCAOP systems, adequate HC intensity is required to achieve reliable spark breakdown through the stable bridging of gas–liquid mixtures across PD gaps. Suitable O_2_ flow rate is also crucial since excessive flow rate can change the PD mode from diffuse to spark to corona and alter the flow mode from bubbly to stratified annular, reducing the PD energy. For the external voltage, positive polarity has less efficiency than negative one due to the lower breakdown field strength at positive polarity and the uneven distribution of density and pressure of the gas/liquid mixture. These rules can be used for similar systems of various sizes.^19^

For wastewater remediation, HC/ED plasma has the advantages of pilot scale, fast degradation kinetics, high energy efficiency (HC bubble reduces the discharge onset voltage), high efficiency even in flow-through mode, no sludge production, no external chemicals (oxidants and catalysts) required, low environmental impact, the in situ formation of different oxidizing chemicals, allowing the ED plasma to propagate through liquids, pH independence, the possible ED-induced pyrolysis of pollutants, simple design (free of additional gas generator and injector), able to inactive bacteria and microorganisms (e.g., *Escherichia coli*.^5^ and viruses^23^), easy operation and higher efficiency than existing cold-plasma-only technologies, while enabling an increase in the energy efficiency of generating ROS in plasma, depending on specific tasks.^3,9,63^ CaviPlasma is reported to have very efficient effects of biocidal and decontamination effects in wastewater.^8,65^ Supercavitation/Cold plasma has been effective in water decontamination and bacteria inactivation.^13^ However, HC/ED plasma has the limitation of moderate-to-high treatment costs, the need for detailed investigations into degradation mechanisms and byproduct formation the prior removal of suspended solids is potentially required (due to orifice plate restrictions), the maintenance of electrodes and hydraulic sections (due to working under pressure rather than suction), etc..^10,12^ Combined HC/plasma for treating microorganisms in water was limited by long treatment times and high energy consumption.^7^ Few works have reported the combination of plasma and bubbles to degrade pollutants.^66^

### 2.4 Mechanism of UC/Plasma

In UC/plasma processes, UC can either cause hot particle collision and ^•^OH production via the H_2_O sonolysis, or provide favorable conditions for stable plasma ignition in UC cavities.^1,35,51^ Tiny UC bubbles in UC/plasma systems can serve as sites in the electrode gap, allowing easier and more effective underwater-plasma generation.^35^ Although spike discharge is required in UC/plasma, the tremendous number of bubbles in cavitation zones can significantly reduce the discharge voltage.^51^ Cavitation zone formation occurs along with cavitation noise, the intensity of which increases with increasing acoustic pressure in liquids. The cavitation noise in fluids during sono-PD does not exhibit a dependence on the electrode material.^2^ Additionally, the mass transfer of liquid to the plasma-cavitation zone is crucial in controlling degradation. In general, acoustic streaming and the flow pattern of forced convection during the circulation of liquid induce mass transfer in liquids.^51^ Acoustic streaming near the ultrasonic sonotrode intensifies liquid mixing and the mitigation of substrates to core reaction zones. Thus, the use of any mechanical stirring (e.g., impellers) can be avoided with large reaction volumes.^35,58^ The flow pattern can be impacted by acoustic streaming and gas injection. For example, Ar injection (8 L/min) in US reactors changed the flow pattern, resulting in slower mass transfer.^58^ Gas injection and Fenton reagents also contribute to UC/plasma treatments. In detail, injecting gas (particularly O_2_ or Ar at large flow rates) in UC/plasma systems improves process performance due to the dissolved gas decreasing the breakdown voltage, favoring the occurrence of UC and plasma. In the case of Fenton reagents (e.g., H_2_O_2_ and ferrous salts), H_2_O_2_ can be produced by UC/plasma itself, ferrous ions can be added from the metal parts of the equipment (e.g., electrodes and container).^1^

UC/plasma processes combine two AOPs, generating numerous microbubbles during UC, significantly expanding the application of plasma for wastewater remediation.^35^ UC/plasma processes are environmentally friendly ways to enhance treatment efficiency without using additional chemicals.^1^ UC has been combined with several types of plasma with differing outcomes. For instance, UC has been combined with high-voltage pulse discharge plasma (also known as ACAP) to ensure stable plasma generation and expand the plasma treatment zone. Plasma channel and pulse discharge occur once large UC bubbles (∼1 mm) are near high-voltage electrodes. Despite the expansion, combination, and accumulation of the UC bubbles, most of them exist in very small sizes. Thus, the extra gas injection in the reaction system can improve the cavitation events and the treatment performance of plasma.^22^ UC has been combined with cold plasma in a flow mode, where UC created an advantageous environment for simultaneous PD throughout the whole fluid.^28^ There are rare reports about the US-assisted activation of RONS in the PAW.^67^ UC effects result in the attainment of critical temperature and pressure conditions, leading to water sonolysis and the generation of free radicals (e.g., ^•^H and ^•^OH), providing a foundation for the reactivation of PAW residual radicals.^68^ However, US-assisted PAW exhibited limited efficacy due to its restricted penetration capacity into aggregated ARB interiors. Although adjusting high-intensity US and free-radical-species concentration in PAW enhanced the bactericidal effects, the low energy efficiency and high temperature limited the use of US-assisted PAW at an industrial level. Catalysts have been used to improve the ROS formation in PAW, but it is challenged by reuse and recycling.^69^ The reactor size for the combination of US and underwater plasma, and the US effects on underwater plasma, is rarely reported.^22,70^ ACAP has been used to improve pollutant removal in wastewater.^35^ A low-temperature US-assisted catalyst-free PAW regeneration process has exhibited excellent decomposition performance with high energy efficiency.^69^ Sequence treatment PAW-SU has been used for inactivating MRSA and MECA.^46^ UC/plasma (sono-plasma) processes were used for flow-mode water treatment. UC/plasma is highly effective in removing the organic dye indigo carmine and *E. coli* disinfection.^2^ Yeasts have been most successfully reduced by high-power sonication at 60 °C after exposure to plasma for 5 min in 2 mL pure culture. Sonication contributed to high temperature, UC, and ROS formation, while plasma also causes UV photolysis and formation of various reactive particles.^14^

Based on the above backgrounds, the differences and similarities between HC/plasma and UC/plasma interactions are summarized in Table 1.

**Table 1:** Differences and Similarities between HC/Plasma and UC/Plasma Interactions

	feature	HC/plasma	UC/plasma
similarities	mechanism of action	both systems use the extreme local conditions generated by the collapse of cavitation bubbles to promote the formation of ROS and to enhance mass transfer	
	physical effects	both generate physical effects, e.g., shock waves, shear forces, and microjets that can physically damage cells (disinfection) or improve mixing	
	plasma synergy	in both systems, the plasma discharge generates additional ROS or RONS. In turn, the cavitation process can enhance the plasma’s effects by improving gas–liquid mass transfer or altering the ED characteristics in the liquid’s flow/bubble field. Cavitation bubbles also favor the propagation of bubbles, further enhancing the mass and energy transfer, and the action of plasma	
	target applications	both are primarily used for advanced water and wastewater treatment (e.g., pollutant degradation and disinfection) due to the powerful combined oxidative and physical effects, but mainly at the lab scale and few cases of wastewater treatment	
	mechanism of degradation	10 3O3+O•H+H+→2O•H+4O2	
		11 H2O→CavitationO•H+H•	
		12 2O•H→H2O+O•	
		13 2H•→H2	
		14 O3→O2+O•	
		15 O•+H2O→2O•H	
		16 $O_{3}+\mathrm{pollutants}\to \mathrm{oxidation}\;\mathrm{products}$	
		17 H2O2→Cavitation2O•H	
		18 O•H+H2O2→HO2•+H2O	
		19 O•H+HO2•→H2O+O2	
		20 HO2•+H2O2→O•H+H2O+O2	
		21 $\mathrm{pollutants}+H_{2}O_{2}\to H_{2}O+{CO}_{2}$	
	process intensification	both systems can be intensified via the appropriate injection of gases (to improve the formation of reactive species, assist the ignition and maintenance of plasma, reduce the discharge voltages, and make up for the shortcomings of insufficient cavitation strength), and the addition of oxidants (e.g., Fenton reagents and H_2_O_2_)	
differences	cavitation generation	induced by the pressure drop when liquid flows through a constriction, leading to a local velocity increase and pressure decrease	induced by intense high-frequency sound waves that cause cyclical pressure variations
	supercavitation	HC-induced stable supercavitation bubble favors the ignition and maintenance of plasma	no similar phenomena are observed
	mass transfer improvement	in addition to the cavitation effects, the fluid circulation also improves the mass transfer. Self-suction effects favor the gas dissolution and the delivery of RONS from gas to bulk liquids	in addition to the cavitation effects, the mass transfer can be enhanced by the acoustic streaming
	bubble characteristics	tends to produce a larger number of larger and less uniform bubble clouds/streamers in a specific geometric region (e.g., vena contracta) with a generally less intense individual collapse compared to ultrasonic systems. Bubble formation is influenced by flow characteristics and reactor geometry	cyclically produces smaller and more uniformly distributed cavitation bubbles throughout the sonicated volume with a more intense individual collapse (higher local temperatures and pressures). Bubble distribution can be less uniform, often concentrated near the ultrasonic horn
	control	controlled mainly by hydrodynamic factors (inlet pressure, flow rate, and reactor geometry/constriction design)	controlled by acoustic factors (ultrasound frequency, power/amplitude, and transducer configuration)
	gas holding	gas holding in HC/plasma systems can be improved via the improved gas–liquid interaction and the self-suction effects, favor the plasma discharge and maintain	sonication can reduce the gas-holding through degassing effects, but it can heat the electrodes to generate additional gas over electrode’s surface
	plasma integration	plasma is often ignited within the cavitation zone or integrated into the flow path. The flow regime is central to the design	plasma is typically ignited above the liquid surface (e.g., DBD) or via electrodes submerged in liquids and exposed to the US field
	operating mode	intrinsically suited for continuous, large-volume flow-mode operation as it relies on fluid flow	can be used in batch or continuous flow, but historically more common in batch or smaller flow systems in research
	mechanical erosion	can lead to significant erosion (damage) to the constriction device (orifice plate or Venturi tube) due to high-velocity flow and bubble collapse	erosion is typically confined to the surface of the ultrasonic horn or transducer
	scalability and energy efficiency	generally considered easier and more cost-effective to scale up for industrial flow-through applications. Often exhibits higher energy efficiency for large-volume processing compared to traditional ultrasonic systems	scaling up to very large volumes can be challenging and costly due to the size and number of transducers required
	application status	relatively many lab-scale cases	relatively few lab-scale cases

### 2.5 Interactions of Cavitation and Plasma

In combined cavitation/plasma systems, the interactions between cavitation and plasma, in most cases, are mutual reinforcement. Synergistic effects have been observed for both the generation and contamination degradation (Figure 4).

**Figure 4:**
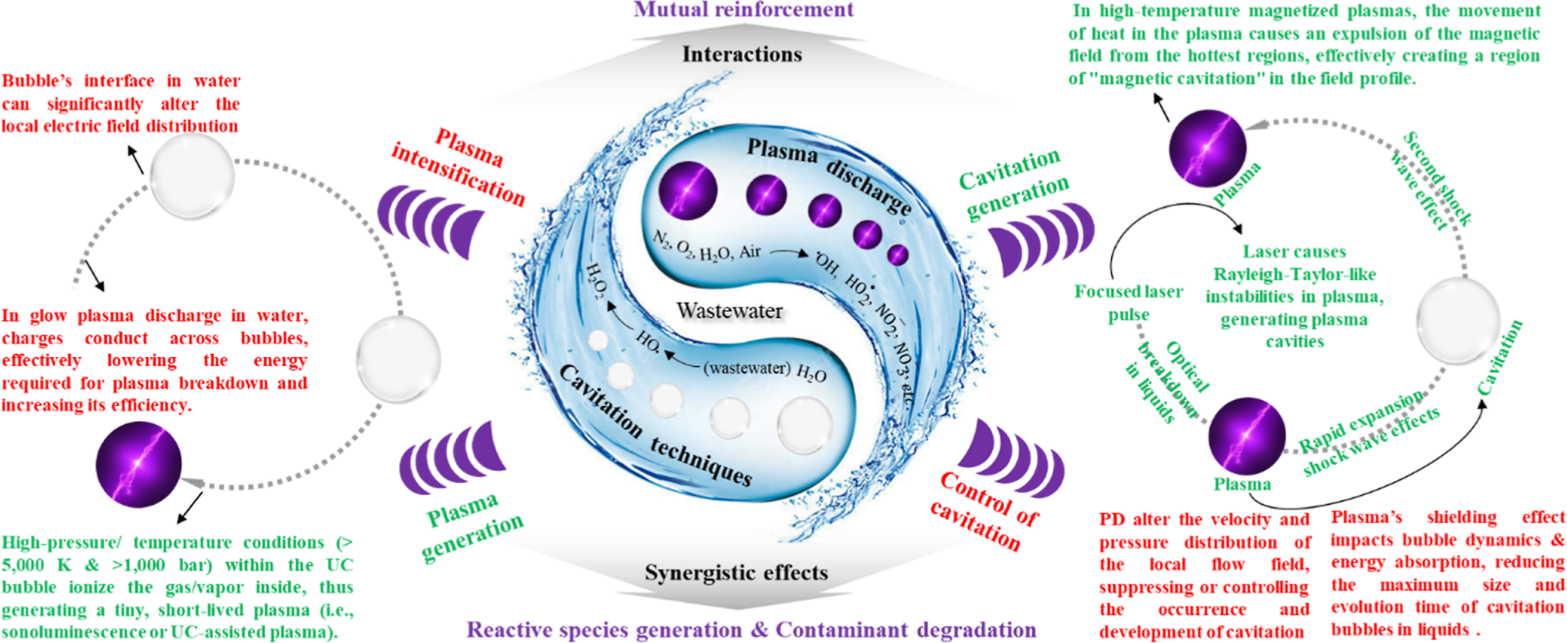
Interactions between cavitation and plasma.

#### 2.5.1 Impact of Cavitation on Plasma

Cavitation favors the generation of PD in the entire volume. Rarified gas is formed inside cavitation bubbles, and, in this gas, it is much easier to produce PD than in liquids.^2^ Both HC and UC can induce plasma generation in cavitation bubbles in a reactor.^5^

In HC/plasma processes, plasma is usually seen as the main influential factor, and HC can assist the plasma.^3^ HC exhibits the highest effectiveness among all developed approaches in providing gaseous conditions in fluids to support the PD.^29^ Unlike the bubbles created by the electrode surface where conduction occurs, HC creates two-phase flow with a gas/liquid mixture via cavitation using orifice plates, Venturi throats, nozzles, etc., creating low gas pressure. Since this is not an electrode process, it contributes to the remarkable reduction in the discharge onset voltage and is ideal for generating discharge plasma.^19^ HC reduces the size of plasma bubbles down to 100 μm and improves gas holdup inside plasma bubbles (11%).^71^ HC-induced spatiotemporally stable supercavitation bubbles do not collapse during treatment, and can thus serve to sustain low-pressure gas atmospheres in bulk liquids, favoring the generation of gaseous plasma and the formation of ROS or RONS.^13^ The generation of plasma at lower voltages may be due to the reduced electric field *E*/*n* (*E*: local electric, *n*: density of medium), which is, in turn, caused by the cavitation bubbles between the electrodes.^5,63^ Generating plasma inside bubbles and then having them collapse in the liquid environment can have a more powerful impact on degrading the pollutants (e.g., Plasma Microbubbles).^3^ The fluid characteristics of HC within a venturi tube are the determining factors for PD properties, the ^•^OH generation rate, and energy consumption in HC/plasma processes. HC governs PD characteristics in HCAOP processes by adjusting the pressures and densities of the gas/liquid mixtures, which influences ^•^OH production rates and energy efficiencies.^19^ HC can assist in plasma treatment since it can produce cavities with larger specific surface areas, followed by better mass transfer of reactive species from gases to bulk liquids and improved RE. These reactive species in gases are self-injected under low pressure into fluids created by HC. Using a given HC reactor, the higher the water flow rate, the greater the pressure drop around the restrictions, and the larger the amount of activated air self-injection into the reaction system.^3^ HC has facilitated the thorough mixing of the highly reactive species in plasma-activated gases in organic-sewage treatment, improving contaminant degradation.^29^ Microbubbles can also improve plasma treatment by activating water. Introducing microbubbles in the plasma system accelerated the degradation rate of dye wastewater by 5-fold.^66^ Suitable O_2_ injection, 3 standard liters per minute (SLPM), increased ^•^OH yield 6-fold up to 119.8 nmol/J due to the alteration in the gas properties (Reactions 10–16). However, owing to the lower breakdown voltage, low O_2_ injection (below 0.5 SLPM) did not improve the ^•^OH yield.^19^ In GPD processes, HC supports the charge conduction through the generated microbubbles.^64^ Plasma-pulse discharges occurred more frequently regardless gas was dispersed or dissolved as tiny bubbles in the electrode gaps.^58^

Unlike HC, UC more complexly impacts plasma processes. Compared to the compression half-cycle of US propagation, pulse discharge occurs more often during the rarefaction half-cycle, since UC cavities are nucleated and quickly expand at low surrounding pressure. Furthermore, prolonged sonication in liquids causes water degassing, gradually reducing the concentration of cavitation bubbles in the interelectrode gap.^58^ UC significantly expanded the possible range for plasma generation in water. Without UC, spark-discharge breakdown occurred only when the solution conductivity was lower than 39.9 μS/cm. With the assistance of UC, the conductivity upper limit for breakdown reached 1002 μS/cm, which was ∼24 times higher than that without UC.^35^ The US irradiation impacts electrical discharge in liquids,^72^ making the PD volumetric in cavitation regions, improving the treatment capacity of UC/plasma.^2^ UC bubbles between the electrode gap can create microchannels, favoring plasma ignition and expanding the electrical conductivity and solute-concentration range for wastewater treatment via plasma. UC assists in a rapid conversion of PD from a low effective streamer discharge to a high effective spark one. Microbubbles are also crucial in the UC-assisted plasma to improve spark PD ignition in liquids. Thanks to the low propagation duration and high energy density in spark PD, more reactive species can be generated, followed by promoted RE.^35^ Enlarged bubbles (>100 μm), in the electrode gaps produced during the rarefaction half-cycle during US propagation and due to bubble accumulation were particularly effective in plasma-pulse spread since such UC cavities favor the free electrons to migrate to high-voltage electrodes and promote the breakdown of liquids.^51^ Acoustic streaming also facilitates plasma processes in both batch and flow modes.^58^ High-frequency waves (27.12 MHz) can heat the electrode, on the surface of which bubbles were generated, meaning that plasmas can thus be easily produced inside the bubbles.^73^

#### 2.5.2 Impact of Plasma on Cavitation

The ignition of plasma in liquids via high-voltage pulse discharge can trigger the generation of cavitation bubbles and US.^74^ Plasma can intensify the HC chain reaction, promoting the ^•^OH formation, and improving the degradation kinetics.^29^ During optical breakdown in water by laser pulses, 15–20% laser energy was converted into bubble energy. Increasing the energy density of plasma elevated the energy converted into shock-wave energy (75% at 40 kJ/cm^3^).^18^ The energy dispersed by the plasma can force the adiabatic expansion of supercritical water inside cavitation bubbles, increasing the bubble diameter up to 2 mm and decreasing their pressure and temperature down to 1 kPa and 50 K, respectively.^74^ Plasma can impact the cavitation bubble dynamics.^17^

### 2.6 Synergism in Cavitation/Plasma Treatment

#### 2.6.1 Degradation of Contaminants

In general, the synergism between cavitation and plasma can be quantified using the synergy coefficient (*S*_c_), which can be calculated using eq 1 ^23,29^. However, the calculation of *S*_c_ values using RE (i.e., *S*_c_ (RE)) instead of the reaction rate constant (i.e., *S*_c_ (*k*)) can also give certain information, but *S*_c_ (*k*) seems more meaningful than *S*_c_ (RE) values. *S*_c_ values can be used to compare the treatment efficiency of different pairs of processes.^46^

22 $$S_{c}=\frac{k_{Cavitation+Plasma}}{k_{Cavitation}+k_{Plasma}}$$

where the *k*_Cavitation+Plasma_, *k*_Cavitation_, and *k*_Plasma_ are the reaction rate constants in the hybrid cavitation/plasma, individual cavitation, and plasma processes, respectively.

It has been reported that the HC/H_2_O_2_ process can verify the synergism of HC and PD in producing oxidant species since this process can simulate the generation rate of ROS. In the HC/H_2_O_2_ process (without plasma), the only source of ^•^OH is added H_2_O_2_. Therefore, adding H_2_O_2_ in a concentration that is similar to the formed ^•^OH level can provide information about the synergism between HC and plasma in forming oxidant species.^64^ The synergistic effects of HC/plasma on wastewater treatment are listed in Table 2.

**Table 2:** Synergistic Effects of HC and Plasma on Wastewater Treatment^e^

wastewaters	plasma	*C*_0_ (mg/L)	*V*_0_ (L)	*t* (min)	*T* (°C)	pH	*P*_i_ (MPa)	*k*_HC_ (min^–1^)	*k*_Plasma_ (min^–1^)	*k*_HC/Plasma_(min^–1^)	*S*_c_ (*k*)	RE_HC_ (%)	RE_Plasma_ (%)	RE_HC/Plasma_ (%)	*S*_c_ (RE)	refs
rhodamine B (RhB)	^a^ED plasma	5.0	5	120	-	3	2	0.0054	0.0034	0.0117	1.69	47.70	34.18	78.03	0.95	^75^
RhB	^b^ED plasma	5.0	5	120	-	3	2	0.0054	0.0034	0.0149	1.32	47.70	34.18	84.57	1.03	^75^
RhB	^c^ED plasma	5.0	5	120	-	3	2	0.0054	0.0034	0.0241	2.74	47.70	34.18	96.86	1.18	^75^
methyl orange (MO)	^d^DBD	5.0	5	30	25–28	8	1.2	0.0002	0.13268	0.18056	1.33	0.78	76.27	89.50	1.16	^23^
TC	ED plasma	50.0	-	-	30	-	7	-	-	-	-	36.40	-	51.70	-	^9^
TC	ED plasma	50.0	5	15	30	-	7	-	-	-	-	36.40	-	98.70	-	^9^
NPX	GPD	80.6	0.02	15			8.7	-	-	-	-			88.17		^64^
metronidazole (MNZ)	GPD	-	-	15	-	-	-	-	-	-	-	14		90.0	-	^64^
E. coli	ED plasma	-	-	-	-	-	-	-	-	-	-	∼10	∼0	95.0	∼9.50	^2^

a Plasma in the storage tank.

b Plasma in self-excited oscillation cavitation.

c Plasma in both the water tank and self-excited oscillation cavitation.

d Plasma in both the storage tank and the cavitation.

e Notes: *C*_0_, initial contaminant concentration; *V*_0_, reaction volume; *t*, reaction time; *T*, reaction temperature; *P*_i_, inlet pressure.

As shown in Table 2, most *S*_c_ (RE) and *S*_c_ (*k*) values are in the ranges of 1.03–9.50 and 1.32–2.74, respectively, revealing the occurrence of synergistic effects in various HC/plasma treatments. It is worth noting that a reaction volume of 5 L is set in most cases, and plasma was introduced in either the tank or the cavitation zones.

The RE*s* of HC/H_2_O_2_ and HC/plasma processes were greater than those of individual processes at 120 min of treatment. Among the various processes, HC/plasma has the highest *S*_c_ as the combination mode can produce more ^•^OH and enhance the mass transfer caused by cavitation.^12,75^ Among HC alone, UV alone, and HC/ED processes, HC/ED in loop mode had the highest RE (100%, 10 min) for treating large volumes (5 L) of FUR (50 mg/L)-contaminated wastewater, even in the presence of other active pharmaceutical ingredients. Only 32% of FUR was degraded by HC alone at 10 mg/L in flow mode, and 43% and 46% of FUR were degraded in 5 and 10 min treatment in loop mode, respectively. Nevertheless, at 10 mg/L, REs reached 100% (*k*_1_ value of 0.8294 min^–1^) using HC/ED plasma for 5 min loop treatment and in flow mode.^12^ Individual HC, DBD plasma oxidation, ultraviolet C (UVC) photolysis, and their combination HC/DBD/UVC were used to treat 5 L of 10 mg/L MO (Figure 5). DBD plasma oxidation exhibited superior MO degradation and treatment capacities (5 L) than individual HC and UVC in MO solutions. UVC alone led to low RE of MO due to its low penetration in dye liquids. In the first 5 min, the degradation rates of MO were HC/DBD/UVC, HC/DBD, DBD/UVC, and HC/UVC in descending sequence. The hybrid HC/DBD/UVC exhibited a significantly higher degradation than other process combinations, achieving an RE of over 91% (16% higher than HC/DBD) for 5 min treatment and an RE of 99.5% over 10 min with a *k*_1_ value of 058,535 min^–1^. For 30 min of treatment, DBD/UVC (92.43%, 0.04560 min^–1^) had higher degradation than HC/DBD (89.50%, 0.18056 min^–1^), while degradation by HC/UVC (4.78%, 0.00159 min^–1^) remained low. Among the various processes, HC/DBD/UVC exhibited the greatest degradation (100%, 0.58535 min^–1^) with a *S*_c_ value up to 4.32. The low efficiency of HC alone may be due to the insufficient ^•^OH generation via HC, but more powerful HC/DBD/UVC could be due to the formation of O_3_, ^•^OH, O_3_^–^, O_2_^+^, O_2_^–^, H_2_O^+^, and H_2_O_2_ via the effects of UC, plasma, and UVC photolysis. HC derived these species sufficiently mixed with MO liquids.^23^ MNZ was degraded by HC, HC/Pulse GPD, and HC/Continuous GPD. HC alone showed lower RE than the various HC/plasma processes. The *k*_1_ value for HC/GPD treatment (0.174 min^–1^) was 18 times higher than that for the individual HC (0.009 min^–1^). The ^•^OH oxidation played the dominant role in individual HC. In various HC/GPD processes, continuous discharge usually exhibited higher RE than the pulsed one. PD had a strong effect on establishing synergy in degrading MNZ. Without cavitation bubbles, there was no PD. UC/plasma processes exhibited similar results. In GPD, the electrical discharge in liquids containing gas bubbles can induce the formation of ^•^OH, H_2_O_2_, atomic oxygen, O_3_, and the emission of UV light. ^•^OH was the major oxidant species in PD. However, due to the short lifetime of ^•^OH (∼3.7 × 10^–9^ s) and its short diffusion in bulk solution (∼6 × 10^–9^ m), most MNZ oxidation occurred at the cavitation–bubble interface. In HC/GPD processes, the high synergism of cavitation bubbles and PD increased the difficulty to evaluate the specific contribution of MNZ removal by oxidation by ^•^OH, photodegradation, and pyrolysis.^64^ Five mg/L RhB dye was treated in wastewater via different AOPs. 33% of RhB was removed via rotor-stator HC at a pilot scale in 60 min in a 15 L solution in a loop configuration (recirculation) in the presence of 75 mg/L H_2_O_2_. 97% RhB was degraded by a hybrid HC/cold plasma in just 5 min at a reaction volume of 5 L and a *P*_i_ of 20 bar in a loop configuration (recirculation). In flow mode at 330 L/h, the RE of RhB was 28% via HC alone and 58% by HC/ED Plasma. Rotor-stator HC/ED plasma treatments were more efficient than individual ozonation, sonolysis, and HC. Similar RE*s* were obtained among HC alone (28%) in flow mode, US/H_2_O_2_ 1:200 (28%), US/H_2_O_2_ 1:100 (27%), 20 min of sonolysis, and 45 min of HC/H_2_O_2_ (25%).^12^ In HC/H_2_O_2_ processes, the RE for MNZ degradation was 23% with a *k*_1_ value of 0.018 min^–1^, while this *k*_1_ value was 9.5 times lower than that in the HC/GPD process.^64^ The RE*s* of RhB using various processes were ordered as HC/plasma > HC/H_2_O_2_ > HC alone.^29^ Plasma alone almost had no effect on a water stream for *E. coli* suppression, while HC/plasma caused a synergetic effect.^2^ Chlorophyll concentration was more rapidly reduced during the treatment of *Microcystis aeruginosa* by HC/plasma, following two or more cycles, than HC alone. In the HC-alone processes, cyanobacterial biomass decreased only slowly, with an obvious effect detectable only after 3–6 days. However, in HC/plasma processes, cyanobacterial biomass was reduced by 95% within a single day. HC impacted cellular surfaces and cell structure (particularly the gas vesicles and subcellular structure), allowing Microcystis to drift. The HC/plasma process led to all gas vesicles collapsing in every cell, thus, which possessed not enough energy to sustain metabolic activity. Cyanobacterial photosynthetic activity significantly changed a day after HC processes, while the impacts were especially obvious after HC/plasma treatment. Compared to the untreated control, Cyanobacterial photosynthetic activity increased after HC and HC/plasma by three cycles. Cyanobacterial photosynthetic activity was considerably reduced 48 and 72 h after the HC/plasma process. The increase in Cyanobacterial photosynthetic activity was probably due to the thylakoid membrane damage by PD. HC-only process slightly stimulated the cyanobacterial primary photosynthetic processes. HC processes only caused the implosion of gas vesicles, but were not targeted directly against photosynthesis. A single cycle of HC/plasma treatment was adequately efficacious to limit photosynthetic function, and complete inhibition of almost all cyanobacterial cells was achieved in the second treatment cycle.^7^

**Figure 5:**
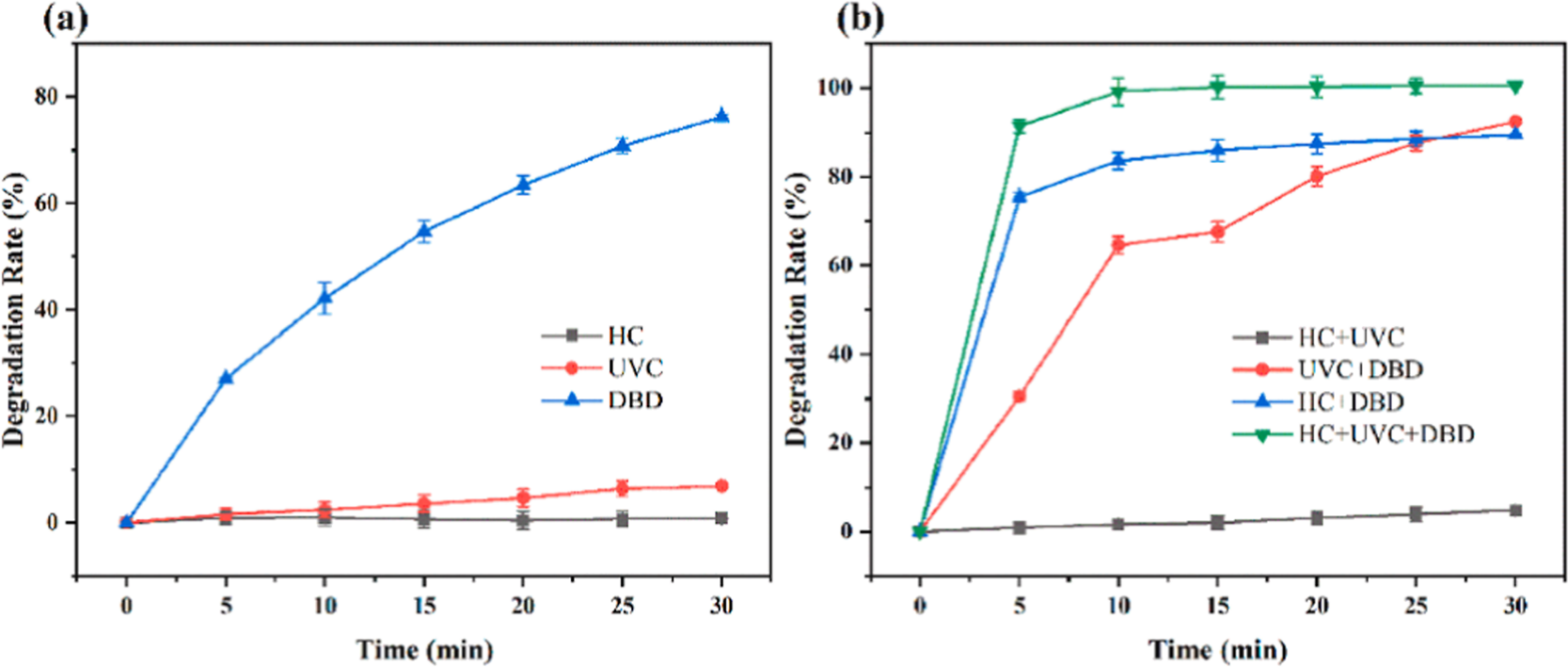
Degradation of MO by various processes. Reprinted from ref 23 Copyright (2024), with permission from Elsevier.

The *k*_1_ values (min^–1^) and RE (%) of various applications of UC/plasma processes for wastewater treatment and water disinfection are summarized in Table 3.

**Table 3:** Synergistic Effects of UC and Plasma on Wastewater Treatment

pollutants	plasma	power (W)	conductivity (μS/cm)	*k*_UC_ (min^–1^)	*k*_Plasma_ (min^–1^)	*k*_UC+Plasma_ (min^–1^)	*S*_c_ (*k*)	RE_UC_ (min^–1^)	RE_Plasma_ (min^–1^)	RE_UC+Plasma_ (min^–1^)	*S*_c_ (RE)	refs
RhB	^a^ACAP	-	20	0.0224	0.0055	0.0301	1.08	23.6	6.4	30.58	1.02	^35^
		-	60	0.0225	0.0013	0.0290	1.22	23.61	1.52	29.38	1.17	
		-	200	0.0224	0.0005	0.0260	1.14	23.60	0.63	26.84	1.11	
	^b^ACAP	-	20	-	-	-	-	23.6	6.4	29.34	0.98	^51^
		-	60	-	-	-	-	23.61	1.52	30.65	1.22	
		-	200	-	-	-	-	23.60	0.63	31.06	1.28	
MRSA	^c^PAW	120	-	0.065	0.035	0.053	0.53	-	-	-	-	^46^
		140	-	0.101	0.035	0.096	0.71	-	-	-	-	
		160	-	0.112	0.035	0.134	0.91	-	-	-	-	
	^d^PAW	120	-	0.065	0.035	0.089	0.89	-	-	-	-	
		140	-	0.101	0.035	0.089	0.66	-	-	-	-	
		160	-	0.112	0.035	0.128	0.87	-	-	-	-	
	^e^PAW	120	-	0.065	0.035	0.105	1.06	-	-	-	-	
		140	-	0.101	0.035	0.169	1.25	-	-	-	-	
		160	-	0.112	0.035	0.149	1.02	-	-	-	-	
	^f^PAW	120	-	0.065	0.035	0.169	1.69	-	-	-	-	
		140	-	0.101	0.035	0.188	1.38	-	-	-	-	
		160	-	0.112	0.035	0.179	1.22	-	-	-	-	

a ACAP without circulation.

b ACAP circulating at a liquid flow rate of 4 L/min.

c The hybrid process is PAW-DU (i.e., PAW with direct US).

d The hybrid process is PAW-SU (i.e., PAW for 5 min following sonication).

e The hybrid process is PAW-SU (i.e., PAW for 10 min following sonication).

f The hybrid process is PAW-SU (i.e., PAW for 15 min following sonication).

As shown in Table 3, the *S*_c_ (RE) and *S*_c_ (*k*) values reach 1.28 and 1.69, respectively. Although the *S*_c_ values in UC/plasma are lower than those obtained in HC/plasma systems, synergism occurs in most cases, suggesting that UC/plasma possesses potential for wastewater treatment. Compared with Plasma alone (0.63%), combined UC/Pulsed discharge plasma radically increased the RE of RhB (31.06%) in flow mode (4 L/min) during treatment of 10 mg/L RhB (Figure 6).^51^ In the US alone, Ar injection at lower gas flow rates remarkably increased the RE of RhB, but high flow rates led to adverse effects. The highest 8 L/min increased the RE of RhB from 30% to 65% for ACAP treatment, from 2% to 50% for individual plasma, and from 20% to 30% for sonolysis alone.^58^ RhB was degraded by ACAP and individual US and plasma at various conductivities. Plasma alone was ineffective, especially at higher conductivity solutions. The RE value of US alone was much higher regardless of the conductivity change. Obvious synergism occurred in ACAP at 200 μS/cm in solution due to the PD being varied from a streamer to a spark-streamer type, producing many more radicals along with more intense physical effects, favoring RhB removal. In plasma-alone, spark PD only occurred at conductivities of <40 μS/cm, whereas the spark PD was remarkably promoted in ACAP processes (particularly at >20 μS/cm). In ACAP processes, increasing the frequency of spark PD also contributes to the synergism of RhB removal. Sonication reduced the thickness of diffusion layers to ≪1 μm, thus increasing the reaction opportunity of RhB with radicals.^35^

**Figure 6:**
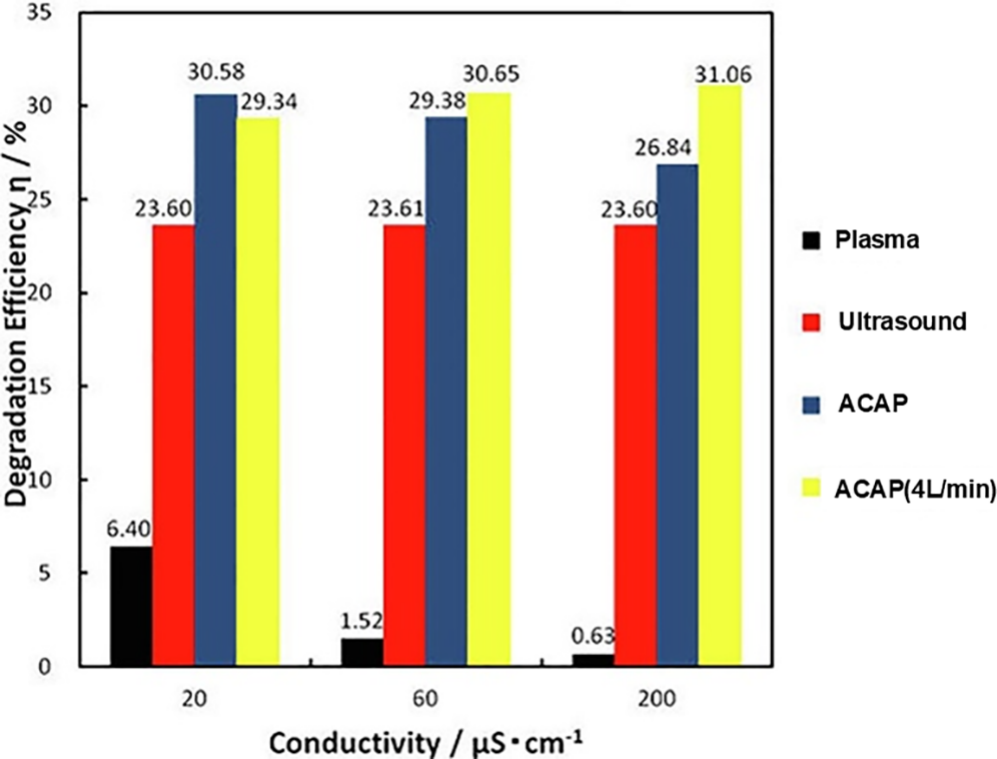
Degradation of RhB by various processes. Reprinted from ref 51 Copyright (2020), with permission from Elsevier.

The inactivation rate of MRSA by various processes was PAW-SU, US, PAW-DU, and PAW in descending order at US intensity of 120 and 140 W; however, at 160 W, the order is PAW-SU, PAW-DU, US, followed by PAW. The inactivation performance of PAW-SU was superior to that of other processes at 120–160 W. PAW (10 min)-SU (140 W) led to 4-log inactivation of MRSA, while the same inactivation can be reached by PAW (>160 W)-DU (15.6 min). Compared to PAW-DU, PAW-SU reduced the US input by ∼1/3. The higher *S*_c_ value of PAW-SU (1.25) than PAW-DU (0.71) can be due to the diffusion of ROS from PAW into the MRSA cell membrane. In PAW-DU, the long duration led to high inactivation. After 15 min of PAW pre-exposure, the overall log inactivation of MRSA with 140 W US treatment nearly doubled from 2.24-log to 4.35-log, while a 1.6-fold increase from 2.89-log to 4.68-log occurred with a US intensity of 160 W.^46^ Typically, gas injection can improve RE, but the extent of the improvement was different in the ACAP, plasma-alone, and US-alone processes, relating to the flow rate, mean, and type of injection. RhB was degraded by various processes with (via an L-shaped nozzle or a porous plug) or without air injection. Regardless of injection methods, air slightly affected RhB removal in the ACAP treatment, but the effect was more obvious in individual plasma processes. For plasma alone, introducing air significantly enhanced RE from only ∼2% (without gas injection) to ∼12% (with gas injection through an L-shaped nozzle). Air injection underneath the sonotrode tip through the glass nozzles led to its dissolution in liquids near the high-voltage electrode. Some air bubbles can approach electrodes because of a lack of acoustic streaming. These effects decreased the breakdown voltage, favoring degradation. The gas injection using a porous plug was generally more effective compared to L-shaped glass tubes. One case demonstrated an RE of ∼33% by ACAP via air injection using a porous plug at 8 L/min, but RE was 28% in the case of glass-tube injection. This gap became even larger in plasma-alone processes where RE*s* were 30% and <12% for porous plug and tube injection at 8 L/min, respectively. Sonication greatly improved RE in ACAP processes, whereas the injection of air was ineffective because it led to the nozzles producing bubbles with relatively large sizes, preventing US propagation and UC-bubble generation. Therefore, Ar was then injected, instead of air, through a more effective porous plug. Ar in water can enhance the ROS generation due to its higher heat capacity ratio, which favors sonochemical reactions. Sonication contributed to RhB removal in ACAP processes more significantly at low Ar flow rates, while the impact of plasma remained significant regardless of flow-rate changes. At the highest Ar flow rate of 8 L/min, the RE via ACAP treatment was 68.0%, and that via plasma alone was 50.6%. The dissolved Ar in water played an increasingly critical role in plasma generation. For the US alone, Ar injection in water at lower gas-flow rates enhanced the RE, whereas negative effects occurred at higher gas-flow rates. This may be due to the injection of gas at high flow rates changing the flow pattern, and hindering mass transfer. In all individual US, plasma and hybrid ACAP treatments, Ar injection was more effective for RhB removal than air injection. In ACAP processes, RE*s* gradually increased with increasing Ar flow rate, while RE was almost unchanged with air injection. The improvement of RE by Ar was especially obvious in plasma-alone treatment and became superior with increasing Ar flow rate. In US-alone processes, RE*s* initially increased and then remained quite stable or slightly decreased with increasing Ar flow rates.^58^ *E. coli* and yeast have been treated in a US-assisted plasma system in submerged and hybrid reactors. For both microorganisms, synergism occurred in the submerged rather than in the hybrid system. In the submerged reactor, synergism occurred without aeration (Supporting Information Figure S1) rather than with aeration. ED can easily occur in US-assisted plasma processes as the electric field inside bubbles may be up to a hundred-fold higher than that found in water. This was probably the main reason for this synergism. On the other hand, the main reason for the disappearance of synergism in a submerged system with aeration was that aerated bubbles exceeded the UC bubbles in number and size. Sonication therefore had almost no enhancing effect on overall inactivation efficiency. The disappearance of synergism in the hybrid system, with or without aeration, may be caused by PD being mainly induced at the tip of the electrode above the water surface, meaning that bubbles contribute less to the PD in the water. Without aeration, a significant difference in the inactivation efficiencies by plasma alone occurred between the two systems, and this might be due to the gas–liquid hybrid-plasma system producing more highly chemically active species (O, ^•^OH, HO_2_^•^, O_3_, H_2_O_2_, etc.) as well as UV on the water surface and in water, and thus performing better than the submerged-plasma system. Moreover, significant decreases in pH and increases in conductivity occurred after individual plasma and US, and combined UC/plasma treatments. For US-assisted plasma treatments, the conductivities were higher than those for plasma-alone treatments. However, the pH decrease during treatment did not impact inactivation efficiency.^22^

#### 2.6.2 Reactive Species Generation

In cavitation/plasma systems, ^•^OH generation via H_2_O decomposition, and derived from e^–^, excited N_2_, and atomic oxygen. The formed ^•^OH can produce H_2_O_2_ through self-combining. H_2_O_2_ formation can thus indicate the ^•^OH generation in plasma systems, the concentration of which in freshwater can reach up to 10^–5^ M, depending on sunlight-exposure conditions.^76^ The ^1^O_2_ formation in gas–liquid PD may be due to the collision of e^–^ with O_2_ in residual air or water, and via dismutation reactions and Haber–Weiss reactions. The gradual increase of H_2_O_2_ and O_2_^•–^ in plasma systems leads to the ^1^O_2_ accumulation over time.^46^ The H_2_O_2_ concentration is crucial to deciding the overall efficiency of cavitation/plasma treatments for contaminant removal.^1^ Even though pure H_2_O_2_ can remain stable for quite a long time, the covalent O–O bond has a low resistance to breakage, meaning that the H_2_O_2_ molecule easily reacts and decomposes under the effect of various substances. The catalyst in the H_2_O_2_ decomposition reaction may be metal ions of variable valency, acids, alkalis, and other random contaminants, some of which inevitably enter the solution. However, the decomposition rate may decrease at relatively low H_2_O_2_ concentrations in liquids.^12^ Moreover, the radical concentration mainly depends on the reaction pH and the presence of salts, etc.^65^

Synergism of reactive species generation may occur in combined processes. Specifically, there was higher oxidant generation in hybrid cavitation/plasma than in individual processes. The concentration of oxidants was stable in HC-alone processes, while it increased in HC/ED plasma systems. For example, the concentrations of oxidants were 0.131 and 1.722 mM for 1 min HC alone and HC/ED plasma treatments, respectively, while the relevant values were 0.153 and 13.426 mM for 15 min treatment. ED plasma can generally linearly increase oxidant concentration over time, with a highest concentration up to 13.426 mM for 15 min treatment.^9^ Similarly, the H_2_O_2_ concentrations for 15 min treatment were 0.12–0.15 and 13.4 mM in HC alone and HC/GPD systems, respectively, and were 87 times higher in a plasma-alone system, with a formation rate of 0.898 mM H_2_O_2_ min^–1^.^64^ Compared with the control Microcystis culture, HC increased the H_2_O_2_ concentration from 3 μM to ∼20 μM, while HC/plasma further increased the concentration to 65–116 μM at various treatment cycles.^7^ During 15 min treatment of 0.3 mM MNZ, only 0.15 mM H_2_O_2_ was formed in HC systems, while 13 mM H_2_O_2_ was formed in HC/GPD systems.^64^ In the HC/DBD/UVC system for MO removal, the O_3_ concentration was 22.4 times higher than that of H_2_O_2_ (0.42 M).^23^ The H_2_O_2_ concentration ranged from 30 to 70 mg/L after 30 min SupCaviPlasma.^8^ H_2_O_2_ (450–580 μg/L/s) and ^•^OH (1.9 μg L^–1^ s^–1^) were dominant species rather than ^1^O_2_ in CaviPlasma systems. Treating by plasma (SIE 45 kJ/L) for 3.5 min, H_2_O_2_ concentration was 84, 70, and 69 mg/L in deionized water, tap water, and ZBB medium, respectively.^65^ At a maximum 8 L/min of Ar injection, the H_2_O_2_ formation rates in RhB solutions were 0.019, 0.083, and 0.111 mg/min/L for single US, single plasma, and ACAP processes, respectively, while the relevant values in pure water were 0.088, 0.031, and 0.127 mg/min/L.^58^

## 3 Application of Cavitation/Plasma Processes

Combined cavitation/plasma processes have been used for the degradation of drugs, dyes, bacterial, and microcystin in water and wastewater, as shown in Figure 7.

**Figure 7:**
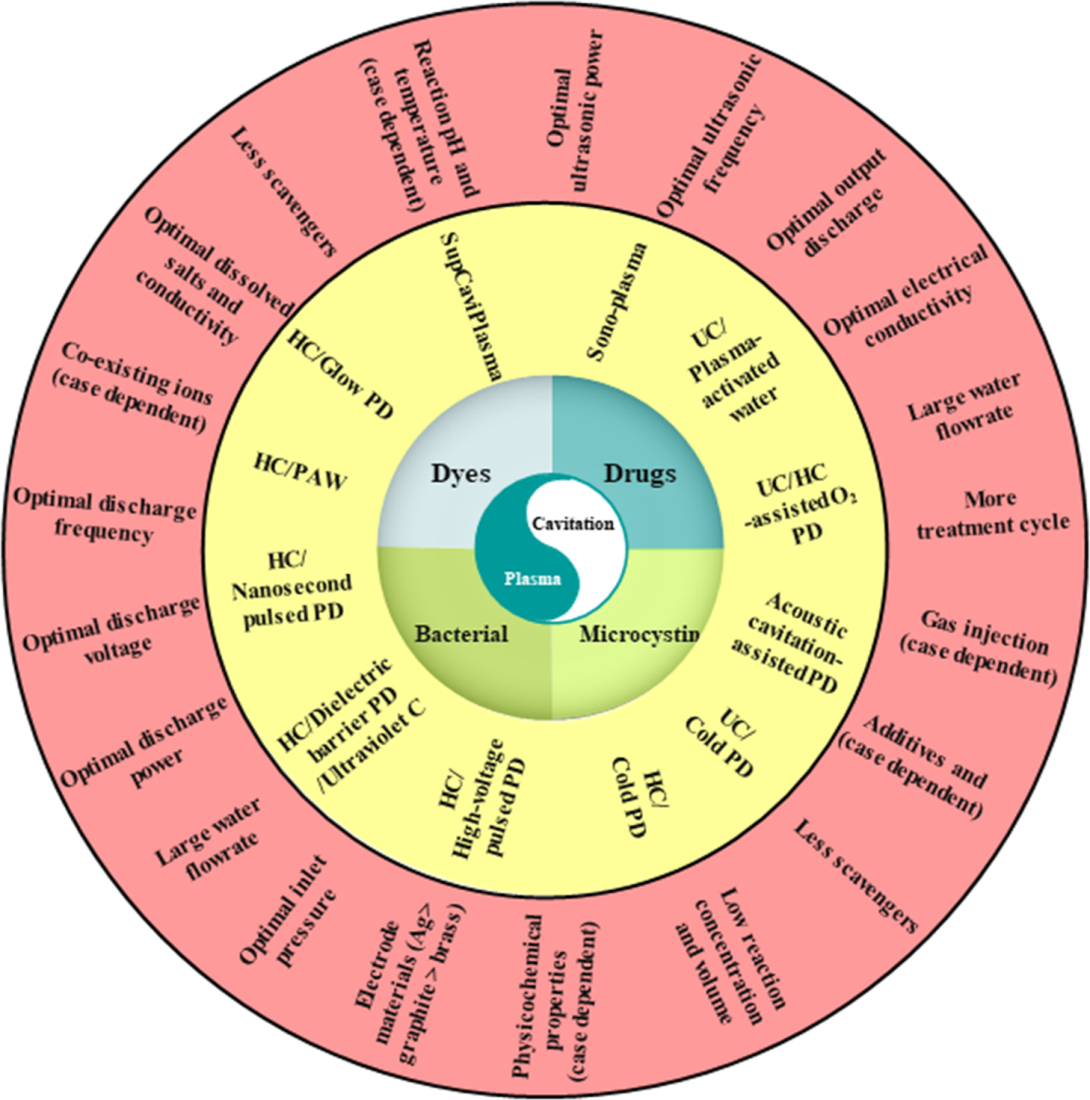
Combined cavitation/plasma processes: application, types, critical effective factors, and favorable conditions.

### 3.1 Application of HC/Plasma

Various HC/plasma apparatuses have been developed, as demonstrated in Supporting Information Table S3. As shown, the developed HC/plasma systems display a variety of properties and capacities. Hybrid HC/plasma systems can be operated in both batch (0.43–20 L) and flow modes (2–50 L/min). Hydrodynamic emitter-, orifice plate-, Venturi nozzle-, converging-diverging nozzle, self-excited oscillation-, and Rotor-stator-based HC reactors have been designed and coupled with DBD, cold plasma, CaviPlasma, SupCaviPlasma, and O_2_ enhanced plasma. For HC, *P*_i_ values ranging from −5 to 250 bar have been tested. In the case of PD, both altering and direct high voltage have been applied, in the ranges of 0.8–50.0 kV, 1–70 kHz, and 24 W-5 kW. Moreover, a range of electrodes has been used, including graphite, silver, brass, titanium, copper, titanium rods, stainless steel rods, tungsten needles, PVC-insulated copper wire, and ring-shaped steel electrodes. Combined HC/plasma has been used for treating pharmaceutical-, dye-, and bacterial-contaminated water, and the main results are summarized in Table 4.

**Table 4:** Application of Cavitation/Plasma for Contaminants Removal in Water

pollutants	conditions	results	refs
tetracycline	5 L of 10–100 mg/L antibiotics were treated in solution by HC/ED plasma on a pilot scale for 15 min at a water flow rate of 330 L/h, 70 bar, 48 kHz, and 30 °C	>98.0%	^9^
doxycycline hyclate		98.0%	
oxytetracycline dihydrate		95.0%	
tetracycline	40 mg/L TC was treated by HC/ED plasma (8.6 kV and 43 kHz) with 5 mg/L catalyst (CeO_2_) in flow mode with no recirculation	41.0% (1st cycle)	^6^
		67.0% (3rd cycles)	
naproxen	20 mL of tap water at an initial concentration of 0.35 mM were treated by HC/PD at a pump pressure of 87 bar, an alternative current voltage of 27 kV with a frequency of 47.5 kHz, and a water flow rate of 2.0 L/min for 15 min	88.17%	^11^
valsartan, tramadol, trimethoprim, sulfamethoxazole, naproxen, diclofenac, 17β-estradiol, propyphenazone, carbamazepine, and bisphenol A	100 μg/L pharmaceuticals were simultaneously degraded by SupCaviPlasma in a Venturi-based lab-scale rotational HC reactor at 53 W plasma power and 25 °C over 30 min	29.0–99.0%	^8^
sulfamethoxazole, norfloxacin, and methylene blue	500 mL of 8 mg/L (or lower) compounds were treated in a Venturi tube-based HC/PAW system and continuous mode (5 L/min) for 30 min	>80.0%	^3^
furosemide and metronidazole	5 L of 20 mg/L (10 mg/L furosemide + 10 mg/L metronidazole) solution was treated by HC/plasma at 20 bar in flow mode	64.0% (furosemide) 43.0% (metronidazole)	^12^
furosemide	5 L of 10 mg/L furosemide solution was treated by HC/plasma at 20 bar in flow mode	52.0%	
furosemide and metronidazole	loop mode for 5 min with the same other conditions	99.0% (furosemide) 93.0% (metronidazole)	
furosemide and metronidazole	loop mode for 10 min with the same other conditions	100.0%	
methanol	7% aqueous solution was treated by HC/plasma at 1000 L/h using graphite electrodes	78.57%	^2^
indigo carmine	100 mg dye was removed from 5 L of 20 mg/L solution within 2 min by venturi-based HC/nanosecond pulsed discharge plasma using negative polarity voltage at O_2_ flow rate of 9.5 L/min	100.0%	^19^
indigo carmine	∼1000 mg/L of dye was treated using HC/plasma	-	^2^
RhB	5 L of 5 mg/L dye was treated by HC/plasma at a pH of 3, a *P*_i_ of 20 bar for 120 min with O_2_ injection at 50 mL/min	97.0% (max)	^23,29^
MO	10 mg/L dye was degraded by HC/DBD/UVC at pH 4, a *P*_i_ of 1.5 MPa, and the utilization of eight UV lamps with a power input of 46.11 W for only 10 min	99.5%	^23^
Salmonella typhimurium	HC/PD (68 kHz) for 17 h	fast decrease	^10^
D. radiodurans	HC/high-voltage pulsed discharge plasma	highly affected	^4^
B. subtilis and S. pombe		slowly affected	
E. coli	HC/PD under continuous flow using silver electrodes	highly suppressed	^2^
Cyanobacterial biomass	HC/Cold PD (400 W)	growth inhibited (3rd cycles)	^77,78^
cyanobacteria	7 days after the HC/PD treatment by one cycle	undetected	^7^
bacteriophage MS2	viruses were inactivated by supercavitation/plasma at >5 log10 PFU/mL in 0.43 L of circulating water after 4 min	>5 log10 PFU/mL	^13^
cyanobacteria	HC/ED plasma after a single cycle	fully inactivated	^79^
E. coli	HC/plasma at a pulse frequency of 6 kHz for 7 μs	95.0% inactivation	^80^
microcystins	48 h after 210 s of treatment by HC/ED plasma	>84.0%	^65^
TC and CIP	24 h following a single cycle of sono-plasma with CeO_2_	52.0% (TC) 41% (CIP)	^6^
RhB	2.5 L 10 mg/L dye was treated by UC/Cold plasma at pH 5, 210 W of US power, 25 kV in flow mode (60 L/h) for 12 min	26.0%	^35^
RhB	2 L of 5 mg/L dye was degraded byUC/ED plasma in batch mode for 12 min at 40 kV, pH 4, and US conditions of 20 kHz and 120 W, with 5 mg/L of FeCl_2_ and 8 L/min of Ar injection	77.0%	^1^
RhB	ACAP process at an electrical conductivity of 61.7 μS/cm and 20 °C	29.38%	^35^
RhB	∼2.5 L dye solution was treated by ACAP at 20 °C and US conditions of 20 kHz and 120 W in flow mode (4 L/min) for 12 min	31.06%	^51^
RhB	5 mg/L dye was degraded via ACAP at 20 °C, 26 kV, and US conditions of 20 kHz and 120 W with the highest Ar injection (8 L/min) for 12 min	65.0%	^58^
lysozyme	UC/HVCAP at 120, 140, and 160 kV for 3 min at a frequency of 120 Hz	obvious inactivated	^81^
E. coli, coliform bacteria, and coliphage*s*	industrial wastewater was treated by sono-plasma	obvious inactivated	^82^
chlorides		100.0%	
hydrogen sulfide		60.0%	
microbiological contamination		68.0%	
methicillin-resistant Staphylococcus aureus	MPAW-US and PAW-US	obvious inactivated	^69^

EY values can be influenced by the concentration and type of contaminants, the characteristics of the hydrodynamic system, and the type of plasma systems, etc.^3,8,83^ The energy consumptions for removing various contaminants are listed in Table 5.

**Table 5:** Energy Consumption for the Removal of Various Contaminants

pollutants	processes	*t* (min)	*P*_i_ (bar)	*V*_0_ (L)	*C*_0_ (mg/L)	RE (%)	*k*_1_ (min^–1^)	cavitation yield (×10^–3^ mg/kJ)	energy consumption (×10^–3^ kWh/m^3^)	treatment cost	EY (mg/kwh)	refs
RhB	HC	120	20	5	5	47.70	0.0054	6.41	216.6	120 ¥/m^3^	23.08	^29^
	plasma	120	20	5	5	34.18	0.0034	1.63	852.1	470 ¥/m^3^	5.87	
	HC/plasma	120	20	5	5	96.86	0.0241	5.92	234.6	130 ¥/m^3^	21.31	
	HC/H_2_O_2_	120	20	5	5	83.10	0.0153	1.02	136.2	110 ¥/m^3^	36.7	
	HC/ED plasma	10	10	5	5	-	-	3.84	7.14	0.44 €/m^3^	13.82	^84^
	HC/ED plasma	10	15	5	5	-	-	3.64	7.33	0.45 €/m^3^	13.11	
	HC/ED plasma	10	20	5	5	-	-	2.76	8790	0.55 €/m^3^	9.94	
MO	HC	30	12	5	-	9.54	-	2.98	-	200.34 USD/m^3^	10.73	^23^
	UVC	30	-	5	-	17.91	-	52.9	-	11.29 USD/m^3^	190.48	
	DBD	30	-	5	-	78.8	-	470.81	-	1.27 USD/m^3^	1694.92	
	UC/UVC	30	-	5	-	15.24	-	5.13	-	116.32 USD/m^3^	18.48	
	UVC/DBD	10	-	5	-	66.45	-	440.92	-	1.35 USD/m^3^	1587.30	
	HC/DBD	10		5	-	83.03	-	74.08	-	8.06 USD/m^3^	266.67	
	HC/DBD/UVC	10	15	5	10	99.5	-	66.14	-	9.03 USD/m^3^	238.10	
E2, BPA, and DF	HC/plasma	-	-	-	-	-	-	0.28–7.22	-	-	1–26.00	^8^
	plasma	10			5	90	-	2.5–7.22	-	-	9–26.00	
FUR	HC/cold plasma	10		5	50	-	-	97.22	-	-	350.00	^12^
indigo carmine	HCAOP	-	-	5	20	-	-	-	0.26	-	-	^19^
MB	HC/plasma	10	-	-	2–10	-	-	1.95–6.37	-	-	7–22.93	^3^
NOR								1.19–3.52			4.28–12.67	
STZ								1.43–3.05			5.15–10.98	
MB	HC/plasma	-	-	-	-	-	-	0.22–0.67	-	-	0.8–2.4	^83^

Compared to plasma in bubbling mode (without liquid flow), the EY (2333 mg/kWh) for 90% RE of MB dye and the volumetric mass transfer coefficient (0.037 s^–1^) in the HC/plasma system were enhanced by 1.63 and 3.08 times, respectively.^71^ The EY value for H_2_O_2_ generation in an HC/Glow-like discharge plasma system was as high as 9.6 g/kWh, and the generation rate reached 2.4 g/h.^85^ The peroxide concentration was >10 mg/L per one water passage through the HC/ED plasma system, and the EY value for H_2_O_2_ generation was 9.5 g/kWh.^86^ EY values for H_2_O_2_ and O_3_ generation by CaviPlasma were about 10 and 0.03 g/kWh in deionized water.^65^ Of the various processes available for RhB removal, the HC/H_2_O_2_ and Plasma alone processes exhibited the highest and the lowest cavitation yield, respectively. HC/plasma provided an excellent cavitation yield.^29^ During TC removal by HC/plasma, the cavitation yield was 8.4 × 10^–5^ mg/J at 60 bar and 8.2 × 10^–5^ mg/J at 70 bar.^9^ The energy deposited to the plasma/the conduction current in water ratio was ∼3% using a 0.7% NaCl solution at a voltage frequency of 10 kHz, which increased to ∼8% at 20 kHz.^5^

Considering the ecotoxicological, mutagenic, and genotoxic effects on plants, animals, and humans resulting from the presence of pharmaceuticals, even in low concentrations, in the environment, and the many limitations due to the low efficiency of wastewater treatment plants, the study and development of new efficient, sustainable, and scalable technologies at an industrial scale is crucial.^12^ The excellent comprehensive performance of the hybrid HC/plasma allows further scale-up to industrial levels.^29^ The above results in the application of various HC/plasma systems pave the way for potential industrial applications. HC/ED plasma is emphasized as a promising intensified and scalable process for purifying water, thanks to its catalyst- and oxidant-free nature, making it suitable for industrial applications.^2,12^ The self-suction of air through the throat and flow system facilitates the extension of the Venturi tube-based HC/PAW system to larger scales.^3^ However, for HC/ED cold plasma systems, although the generation of ^•^OH, ^•^H, ^•^O, and H_2_O_2_ has been demonstrated, the fact that their setup design favors the generation of plasma within bubbles places significant constraints on its scalability.^12^

### 3.2 Application of UC/Plasma

Unlike HC/plasma, few UC/plasma systems have been developed for wastewater remediation (Supporting Information Table S4). As shown in Table S4, the various UC/plasma systems are able to work in both batch and flow modes (1.0–16.7 L/min). The used US frequencies and powers are in the ranges of 0.3–60.0 kHz and 140–330 W (1.5–15.5 W/cm^2^), respectively. The used voltages are in the ranges of 8.6–32.0 kV and 25–43 kHz. Unlike UC/plasma systems, hydrodynamic emitters have been used to introduce acoustic energy into fluids in UC/plasma systems. Combined UC/plasma has been used for treating antibiotic-, dye-, lysozyme-, and microbiological- contaminated water, and the main results are summarized in Table 4.

The optimal frequency of electrical pulses (68 kHz) ensured the lowest electrical energy consumption while maintaining the desired efficiency. The power consumption of a sono-plasma at 68 kHz was 2.5 kW·h/m^3^, which was remarkably lower than that of traditional purification by ozonation, where 13.2–30.2 kW·h power was required to achieve a similar result. Electricity accounts for the largest cost, making the cost-efficiency of sono-plasma exceed ozonation by 5–10 times for water treatment.^82^ The energetic efficiencies of ACAP and US alone without Ar injection were 0.11 and 0.08 g/kWh, respectively. Injecting Ar at 8 L/min, the energetic efficiencies of ACAP, Plasma alone, and US alone were 0.26, 1.98, and 0.13, respectively.^58^

The developed treatments have great potential in wastewater treatment. Nevertheless, despite having positive laboratory results, the majority of cases cannot be used on an industrial scale.^58^ High flow rates of the sono-plasma treatment make this method potentially useable at industrial levels.^2^ A lab sono-plasma reactor shows great scalability and can be hybridized with HC in flow mode at industrial scales.^6^

## 4 Effect of Critical Factors in HC/Plasma Processes

### 4.1 Contaminant Concentration and Reaction Volume

Generally, low initial concentration and reaction volume favor the degradation. For example, 10–100 mg/L TC solutions were treated by HC/ED plasma at a *P*_i_ of 70 bar. The best RE*s* were achieved at 10 mg/L, especially 66.5% in flow mode and 99.5% after 5 min batch treatment.^9^ After treatment for 15 min by HC/PAW, the RE was 75.4% in a 2 L, 2 mg/L MB solution, while RE*s* were 50.76% and 33.77% at high volumes of 10 and 20 L, respectively.^3^

### 4.2 Reaction pH and Temperature

pH values in most wastewater discharges are in the range of 4–9.^64^ MO was degraded fastest at a pH of 4 by HC/DBD/UVC in solutions. The quinone structure in MO at acidic pH makes it more susceptible to degradation by HC/DBD/UVC, which promotes the generation of ^•^OH among other particles.^23,87–89^ Decreasing pH facilitated MNZ degradation by HC/GPD in drinking water.^64^ pH had no obvious influence on the removal rates of TC, DC, and OC by HC/ED.^9^ Increasing pH from 4 to 9 gradually decreased the total residual oxidant concentration from 19.1 to 8.34 mg/L. H_2_O_2_ was undetectable at pH 4, but its final level increased up to 274.3 μM with continuous increases in pH value. Increasing pH increased the accumulated ^•^OH concentration from 24.8 to 59.7 μM.^8,19^ When plasma was applied at industrial levels, the pH decrease was not crucial as the wastewater constituents acted as a buffer and mitigated this impact. Nevertheless, when plasmas were introduced for surface or drinking water, slight pH reductions were beneficial. For instance, the pH drop in SupCaviPlasma was generally more conservative than that in atmospheric-pressure plasmas (2.0–3.5).^8^

Ten micropollutants were processed by HC/plasma for 0.5 h at 15–60 °C in a SupCaviPlasma device (Supporting Information Figure S2). The highest RE reached 99% for E2 and BPA, followed by DF (89%), while the REs of the other compounds were lower. The RE*s* of VAL and NPX were 63%, while the RE*s* of PPZ, TMP, SFX, and TMD were ∼40%. Compound removal depends on sample temperature. For most micropollutants (E2, BPA, NPX, PPZ, CBZ, TMP, SFX, and TMD), REs decreased with increasing water temperature, and the lowest RE*s*, of 9–93%, were reached at 60 °C.^8^

### 4.3 Scavengers, Dissolved Salts, and Conductivity

Inorganic anions (e.g., HCO_3_^–^ and Cl^–^) and some organics (e.g., alcohols and solvents) in liquids can quench ^•^OH and alter conductivity, decreasing RE. The mean concentrations of HCO_3_^–^ in tap water are 200–500 mg/L. Interactions of CO_3_^2–^ and HCO_3_^–^ with ^•^OH promote the CO_3_^•–^ generation (Reactions 22–24). CO_3_^•–^ has a lower oxidation potential (1.63 V) than ^•^OH (2.34–2.70 V). Increasing salt concentrations also decreases cavitation bubble size and inhibits bubble accumulation. Due to the salting-out effect, electrolytes also decrease gas solubility in liquids, influencing the levels of active bubbles. Electrolytes also affect the surface tension and viscosity, which can affect cavitation bubble formation and implosion in liquids. The presence of inorganic constituents in aqueous solution can also influence PD. Explaining the single effect of inorganic ions in solution in PD is difficult due to strong synergistic effects. EtOH can scavenge ^•^OH radicals (Reaction 25) due to the relatively high volatility of EtOH (vapor pressure = 55 mmHg), which favors its diffusion into cavitation bubbles during their formation, quenching radical-induced oxidative interactions at gas–liquid interfaces. *t*-BuOH can quench ^•^OH both in the gas phase and at the gas–liquid interface of the cavitation bubble. Changes in solution conductivity alter charge conduction during PD. In a high-conductivity solution, the breakdown process required for PD is hindered due to the ion flow, which causes electric field polarization.^12,64,90^ The emission intensity of ^•^OH and ^•^O in plasma in a cavitation field decreases as conductivity increases at a constant electrical power supply, due to the decreased power deposited for plasma production.^5^

23 CO32−+O•H→CO3•−+OH−⁣k=4.0×108mol−1s−1

24 HCO3−+O•H→CO3•−+H2O⁣k=1.0×107mol−1s−1

25 HCO3−+O•H→OH−+HCO3•

26 CH3CH2OH+O•H→C•H3CH2OH+H2O⁣k=3.1×1012cm/mol/s

For example, the RE of MO in solution was 94% when using HC/DBD/UVC with the addition of 100 mL of *t*-BuOH for 10 min, while the RE was only 82.3% with the addition of 500 mL of *t*-BuOH (Supporting Information Table S5).^23^

Adding 2 and 4 mM HCO_3_^–^, the *k*_1_ values of MNZ removal by HC/GPD were 0.112 and 0.112 min^–1^, respectively. Solution conductivities changed from 896 × 10^–6^ S/cm with 2 mM HCO to 1812 × 10^–6^ S/cm with 4 mM HCO_3_^–^. Upon adding 0.45 and 2.00 mM Cl^–^, the *k*_1_ values of MNZ removal were 0.095 and 0.099 min^–1^, respectively. Solution conductivity ranged from 349 × 10^–6^ S/cm with the addition of 0.45 mM Cl^–^ and 1551 × 10^–6^ S/cm with the addition of 2 mM Cl^–^. Compared with treatment using distilled water, the *k*_1_ values were 1.55 times lower in the case of HCO_3_^–^ and 1.84 folds lower for the case of Cl^–^.^64^

The ^•^OH-accumulation concentration in HC/nanosecond pulsed discharge plasma varied upon increasing the conductivity (0.52–6.76 S/m). At <1.43 S/m, the ^•^OH level remained ∼26.17 μM regardless of conductivity changes. Further rises in conductivities, the ^•^OH level suddenly declined to 4.21 μM at 2.37 S/m, and then gradually decreased until it ceased to form at ∼7 S/m. At >7 S/m, the voltage failed to load across the PD gap and was thus unable to generate discharge. At <2.37 S/m, the power consumed in the PD gap intensely increased with increasing conductivities from 31.2 to 95.3 W. At high solution conductivity (≥1.42 S/m), both the energy efficiency and ^•^OH production rate in HC/plasma systems severely decreased.^19^

### 4.4 Discharge Frequency, Voltage, and Power

Adjusting PD power, voltage, and frequency in cavitating water can tune the generated ROS (e.g., H_2_O_2_) concentration.^10^ For instance, increasing the ED amplitude frequencies from 10 to 48 kHz in pilot-scale HC/ED plasma processes increased TC (50 mg/L) degradation, where the maximum RE*s* were 96.8% and 98.7% at 10 kHz and 48 kHz for 15 min treatment at 70 bar, respectively.^9^ The disinfection efficiency of *E. coli* by HC/PD was positively related to total reaction time and discharge pulse duration, but was insensitive to pulse frequency.^80^ The RE*s* of DF using the plasma-microbubble technique increased with an increase in the PD voltage from 4 to 6 kV, and decreased at 8 kV due to charge gathering and discharge variability, followed by a reduced generation of ROS.^23,26^ BPA, DF, E2, and PPZ were treated by HC/Subatmospheric-pressure plasma at 25 °C and various discharge powers (25–53 W) for 30 min (Supporting Information Figure S3). The RE*s* increased with the rising powers of plasma for all model compounds. When the power was doubled, the RE increased >6 times for BPA, 4 times for DF, 5 times for E2, and ∼1.5 times for PPZ.^8^

### 4.5 Inlet Pressure and Water Flow Rate

The initiation, magnitude, and intensity of cavitation all relate to the reaction pressure and *C*_v_.^23,91^ Generally, higher *P*_i_ values in HC reactors lead to larger pressure drops, enhancing cavitation bubble generation, causing a greater amount of ^•^OH production, and higher oxidative degradation of organics.^92^ Increasing *P*_i_ values can increase water flow rates in HC reactors and decrease *C*_v_ values. Smaller *C*_v_ numbers suggest more bubble implosion, leading to greater shear zones and intensified turbulence in HC reactors, thus intensifying HC impacts. Decreasing the number of cavitation bubbles may induce bigger bubbles, thus weakening HC impacts.^23^ Increasing the *P*_i_ of HC reactors can also slightly increase the size of HC bubbles, burst pressure, temperature, energy, turbulence intensity, and H_2_O pyrolysis, and decrease pressure losses, enhancing ^•^OH generation followed by increased pollutant RE*s*.^93^ Increasing water flow rates can, in turn, decrease pressure in the restriction unit.^3^ More intense HC effects occur at very high fluid velocities or very low *P*_i_ values.^23,91^

For example, 5 L of 5 mg/L RhB solution was treated using HC/ED plasma on the pilot scale and at *P*_i_ of 10, 15, and 20 bar. HC/ED plasma led to the highest RE of 58% at 20 bar. RE was decreased at low *P*_i_ of 15 bar (52%) and 10 bar (39%) in flow mode, while it was improved in a loop process. In loop HC/ED plasma, the RE was 97% at 20 bar for 5 min of treatment, 98% at 15 bar for 10 min, and 94% at 10 bar for 10 min. The rise in *P*_i_ during HC/ED plasma also increased *k*_1_ values, and the highest *k*_1_ value of 0.6598 min^–1^ was achieved at 20 bar.^12^ 500 mL of 2–10 mg/L STZ and NOR solution was treated in a Venturi tube-based HC/PAW system at circulated water flow rates of 3, 4, and 5 L/min. At flow rates above 3 L/min, more contaminants were degraded with higher *k*_1_ values. The RE*s* of 5 mg/L at flow rates of 3, 4, and 5 L/min were 36%, 50%, and 63% under the same remaining conditions, respectively, while the relevant RE*s* of 2 mg/L were 48%, 81% and 86%. The gaps in the RE*s* at various flow rates at 8 and 10 mg/L are insignificant. At flow rates of 3, 4, and 5 L/min, *k*_1_ values were 0.011, 0.121, and 0.180 min^–1^ at 5 mg/L, respectively, and the relevant *k*_1_ values were 0.017, 0.056, and 0.067 min^–1^ at 8 mg/L. For 10 mg/L NOR, the *k*_1_ values were 0.038, 0.063, and 0.072 min^–1^ at 3, 4, and 5 L/min, respectively. Increasing the water flow rate in the HC/PAW system enhanced RE by improving HC and the self-suction of more ROS in the fluids. More ROS can enter the HC chamber at higher water flow rates.^3^

### 4.6 Physicochemical Properties and Electrode Materials

HC/subatmospheric-pressure nonthermal plasma has provided a range of different RE*s* for various micropollutants (Supporting Information Figure S4). These differences are probably due to the pollutants’ various physicochemical natures such as Henry’s law coefficient (indicates the volatility), log*K*ow (indicates the hydrophobicity), and p*K*_a_ (indicates the ionization degree of a compound at given pH) values, which determine the distribution of the substrates (near the bubble interface or in the bulk liquid) relative to the ROS (bubble interfaces).^3^

A suspension of *E. coli*. (2.5 × 10^8^ per mL) was treated via HC/plasma using silver, graphite, and brass electrodes. The best inactivation was observed using silver electrodes, and brass electrodes led to the lowest inactivation (Supporting Information Figure S5). The inactivation of *E. coli* reached 98% after treatment by HC/plasma with silver electrodes, which can result from both the silver ions and UC, free radicals, O_3_, and UV light. The observed prolonged oxidation may be induced by H_2_O_2_ and oxides of metals.^2^

## 5 Effect of Critical Factors on UC/Plasma Processes

### 5.1 Reaction pH, Temperature, and Conductivity

The composition of the ROS in the various AOPs in liquids mainly depends on pH instead of the means of H_2_O breakdown.^6^ Radical ^•^OH can dissociate in strong alkaline solutions (^•^OH + OH^–^ = O^–^ + H_2_O or ^•^OH = O^–^ + H^+^).^51^ Specifically, low pH (3–5) favors RhB degradation.^1,35,51^

REs of RhB have been observed to remain stable regardless of temperature control, as both UC and plasma led to thermal effects in electrode gaps, spreading heat in the whole system. The degradation reactions of RhB all occurred in the electrode gaps, where the temperature could be extremely high, regardless of cooling.^1^

PDs are sensitive to solution conductivity.^22^ For instance, increasing conductivity (10–20 μS/cm) severely increased RE*s* and degradation rates of RhB to the maximum in ACAP systems, and then degradation slowed with a further increase in conductivity (Supporting Information Figure S6).^35^

### 5.2 Ultrasonic Power, Frequency, and Output Discharge

The RE was almost unchanged when increasing US power from 210 to 280 W in the treatment of 10 mg/L RhB by UC/Pulsed discharge plasma at pH 5.^35,51^ US amplitude had an insignificant influence on RhB removal by ACAP, and this may be due to the extremely high UC intensity and perhaps near saturation even under the minimal US amplitude. In this case, cavitation characteristics may remain unchanged regardless of variations in amplitude.^35^ At higher output voltages in ACAP systems, both RE and rate constant increased due to higher output voltage, resulting in greater electron level and higher amounts of ROS (e.g., ^•^OH, HO_2_^•^, and H_2_O_2_), attacking RhB molecules. Higher voltage output also enhanced physical effects, assisting RhB degradation. Higher RE values at voltages of 28–32 kV implied that the spark discharge offers better removal.^35^

### 5.3 Contaminant Concentration, Water Flow Rate and Treatment Cycle

2.5–10.0 mg/L RhB was treated by ACAP at 25 kV, pH 5, and 200 μS/cm for 30 min. Increases in initial RhB concentration decreased both RE*s* and *k*_1_ values.^35^ Increasing initial RhB levels by 4 times caused a 2.3-fold increase in RE in UC/Pulsed discharge plasma systems.^51^

An increase in liquid flow rates (2–8 L/min) increased the RE*s* of 10 mg/L RhB from ∼19% to 26% in ACAP processes at 20 μS/cm, US power of 210 W, 25 kV, and pH 5 .^51^ After the first cycle (5 ms) of sono-plasma in flow mode, the RE*s* of TC and CIP were 41% and 25%, respectively, and relevant RE*s* were 67% and 55% after three treatment cycles.^6^

### 5.4 Gas Injection

RhB was degraded by ACAP with either Ar or air injection into the bath via L-shaped nozzles or a porous plug. The RE of RhB was almost unchanged with air injection, but doubled with Ar injection. Compared with air, Ar injection can obtain higher temperatures during UC, inducing more intense generation of chemically active O and H-containing ions and radicals, highly reducing the breakdown voltage, increasing the frequency of plasma pulse discharge (>30 Hz) at a constant working voltage, increasing solubility in water (molar fraction of 2.748 × 10^–5^ for Ar and 1.524 × 10^–5^ for air), lowering entropy, and avoiding the production of undesired particles that limit the PD and RhB removal. RE can decrease in ACAP processes with inducing air, particularly at lower flow rates, since glass nozzles generate bigger bubbles. These bubbles can escape from cavitation zones before collapse, without reaching the high-voltage electrode. Moreover, N_2_ can be oxidized by O_2_ under the extremely high temperatures created by UC and plasma to form NO_2_ and NO. ^•^OH can then be trapped by them to produce soluble nitrite or nitrate ions (Reactions 26 and 27). This increases solution ionic conductivity, reducing the pulse frequency of PD due to the ionic current occurring in the electrode gaps, increasing the breakdown voltage. The RE of RhB thus reduces because of the tapping of ^•^OH and the increase in breakdown voltage with air injection.

27 NO2+O•H→H++NO3−

28 NO+O•H→H++NO2−

Gas injection via porous plugs is generally more efficient than via L-shaped nozzles as porous plugs produce tiny bubbles, which slowly rise in the solution bath (e.g., at 8 L/min, ∼2–3 mm bubbles were generated with a low rising velocity of 0.2 m/s), allowing them to reside in water much longer than big bubbles. Thus, bubbles can enter fluids in the reactor and approach electrodes easily. Although the tiny gas bubbles generated using porous plugs are unable to enter UC zones in electrode gaps due to the large gradient of sound pressure, they can enter the circulatory flow. This also prolongs their residence in liquids, favoring gas dissolution. Moreover, tiny bubbles possess high contact areas, favoring gas dissolution again. The generation of tiny bubbles, high contact area, long residence duration, and improved gas dissolution all contributed to the improved RE.^58^ Ar injection (7.2 kV/cm) gives a slightly lower breakdown voltage than air injection (35.5 kV/cm) in ACAP processes. These are different from those observed with gas injection in water, since both air and Ar exist in water either in microbubbles or in the dissolved state in small amounts. The difference in the mean frequency of PD between air and Ar injections became more obvious when plasma was generated without sonication, especially with injection interruption. There were no bubbles in the electrode gaps after interrupting gas injection. However, tiny bubbles in other zones of water impacted the discharge frequency. Thus, it was difficult to distinguish the effects of the dissolved gases and bubbles on the breakdown voltage and discharge frequency. The dissolved gas and its properties played major roles in improving RE.^35^

### 5.5 Additives and Scavengers

TC and CIP were degraded by sono-plasma with CeO_2_ or co-existing ions. Adding CeO_2_ favored the sono-plasma by inducing sono-catalysis and photocatalysis. Moreover, sonication can mitigate the aggregation of CeO_2_ nanoparticles, improving catalytic activity. Anions can be adsorbed onto the surface of CeO_2_, reducing the adsorption capacity and catalytic activity of CeO_2_ due to the filling of pores at the catalyst surface. Anions (carbonates and nitrates) can act as radical scavengers, quenching free oxidizing radicals (e.g., ^•^OH), and inducing the generation of less oxidative species, decreasing degradation. However, this contributed to prolonged oxidation (up to 24 h) due to the formation of long-lived species. Hardness ions, e.g., Ca^2+^ and Mg^2+^, can reduce the efficiency of sono-catalysis. Adding Fe^2+^ triggered Fenton-like reactions, which can be enhanced by light and US irradiation in the sono-plasma process. It was possible to regenerate Fe^2+^ via the photolysis of Fe^3+^.^6^ FeCl_2_, a square iron plate, and a stainless-steel sonotrode can serve as Fe^2+^ sources in ACAP systems to support the RhB removal via Fenton-like reaction.^1^ Additionally, adding TBA and l-histidine (l-His) in PAW, PAW-DU, and PAW-SU systems significantly decreased the inactivation of MRSA cells, suggesting the critical roles of ^•^OH and ^1^O_2_ in PAW-SU treatments.^46^

## 6 Environmental Impactions and Future Perspectives

As the Ag^+^ concentration exceeded permitted limits, silver electrodes cannot be used in HC/plasma processes for treating drinking water.^94^ HC/plasma treatment at flow-through mode maintained the integrity of cells and avoided the release of cyanotoxins and other organics. HC/plasma processes can be completed within seconds in a single treatment, avoiding prolonged or repeated treatments, and releasing potentially hazardous microcystins.^95^ Water treated with cold plasma within a stable supercavitation bubble for a short time successfully inactivated a virus and did not pose cytotoxic effects toward in vitro HepG2 cell model systems or adverse effects on potato-plant physiology.^96^ Removing the cyanobacterial biomass gently before treating cyanobacterial water blooms with HC/plasma avoided cell destruction and cyanotoxin release.^95^ HC/plasma offered high-volume throughput and was free of gas or chemical additives, and the pH of the outlet did not require adjustment after treatment, making the process environmentally friendly.^95,97^ Utilizing electrons as a green reagent for the in situ formation of highly reactive ^•^OH in plasma processes is an environmentally friendly approach for degrading organic pollutants. In situ H_2_O_2_ production prevents dangerous handling and storage in industrial facilities.^98^ However, introducing catalysts in cavitation/plasma processes can generate secondary pollutants and lead to their residues entering water environments, impacting living organisms.^3^

Combined HC/plasma and UC/plasma represent promising AOPs, particularly for water and wastewater remediation. This hybrid approach leverages the unique mechanical, thermal, and chemical effects of both phenomena to achieve synergistic process intensification.^100–119^

Compared to the individual technologies, the primary potential of HC/plasma and UC/plasma lies in the synergistic effect that significantly enhances the production of reactive species and overall treatment efficiency. In combined systems, cavitation (especially HC) produces localized “hot spots” with extreme temperatures and pressures, generating powerful oxidizers (e.g., ^•^OH, H_2_O_2_, O_3_, and ^1^O_2_), while plasma further boosts this by introducing intense UV radiation and charged particles, with the cavitation bubbles acting as a favored medium for plasma discharge. Meanwhile, cavitation triggers microturbulence and shockwaves, enhancing mixing and mass transfer, overcoming a common limitation of individual plasma or other AOPs where the pollutant needs to move to the reaction site. These enable the powerful process intensification, leading to much higher RE of refractory pollutants (e.g., pharmaceuticals, dyes, pesticides) and superior disinfection performance. Combined processes can achieve high RE without the continuous addition of external chemical oxidants or catalysts, reducing operational costs and sludge generation. Nevertheless, in terms of scalability, HC/plasma is generally more energy-efficient and easier to scale up than UC/plasma.

Even though the great potential, several technical and economic hurdles (e.g., energy utilization rate, reactor design and optimization, mechanism clarification, high initial investment, and equipment longevity and maintenance) need to be overcome for widespread adoption. Accordingly, the overall energy efficiency of the hybrid systems is still a significant bottleneck; thus, optimizing the energy delivery to maximize synergistic effects is crucial. Additionally, critical factors like inlet pressure, flow rate, electrode configuration for plasma, and the geometry of the cavitation device are interdependent and difficult to rationalize and standardize, making the difficult to develop robust, efficient, and scalable hybrid systems. Mechanisms, especially the complex and transient interactions between the collapsing bubbles, plasma formation, and radical chemistry, still require further to be clarified. As we all know, cavitation can cause erosion (material wear) on reactor surfaces, especially on the constricting parts (Venturi or orifice plates). Integrating plasma electrodes into this harsh, turbulent environment can further complicate equipment lifespan and maintenance. Advances in materials science will help mitigate the erosion issues.

Despite these challenges, the outlook for cavitation–plasma combinations should be highly positive, driven primarily by the urgent need for efficient and sustainable solutions for environmental remediation. The immediate and most realistic application is in wastewater and drinking water treatment, particularly for eliminating pharmaceuticals and endocrine disruptors, as well as for industrial dye degradation and disinfection. Future research is expected to focus heavily on reactor design optimization to increase energy efficiency by precise control over the cavitation cloud and discharge location, and scalability. Using real wastewater instead of synthetic wastewater and assessing and controlling the possible toxicity of generated oxidation products will be of great interest to all sides. The hybrid technology will likely be integrated as a pretreatment or polishing step within a multibarrier system or AOPs. For instance, improving the treatment effect by ozonation after HC/plasma treatments, and testing process suitability for treating other wastewater types. The optimization and extension of the microbubble-enhanced cold plasma activation system using other chemicals and gas injection. Applying the hybrid process to generate H_2_O_2_ in situ and then coupling it with a low-cost Fenton process or photocatalysis. Moreover, it is essential to test the combined treatments for water remediation in African regions involving local organizations dealing with water treatment. Beyond water and wastewater treatment, as the technology matures, applications may realistically expand into other fields requiring intense localized chemical or mechanical effects, such as chemical synthesis, food processing (e.g., extraction, sterilization), and biofuel production.

## 7 Conclusion

Combined HC/plasma and UC/plasma support wastewater treatments through their unique merits, like being free of additional additives, fast processes with satisfactory outcomes, and maintaining biocell integrity while avoiding cytotoxin release, prolonged oxidation, and so forth. Powerful treatment ability and great outcomes are, in many cases, the synergistic effects between cavitation and plasma techniques, which is especially true when running at optimized conditions. Combined cavitation/plasma processes allow the degradation of contaminants in water and wastewater more cleanly, efficiently, and economically compared to the conventional strategies, thanks to the less use of chemical reagents, the diverse ROS sources, and shorter running duration.

## Supplementary Material



## Notes

Possible mechanism of synergistic effect with US-assisted plasma treatment in microorganism inactivation; removal of investigated micropollutants (100 μg/L of each) after 30 min of SupCaviPlasma treatment at different sample temperatures; removal of BPA, DF, E2, and PPZ during plasma–cavitation treatment (25 °C) at different plasma powers; change in absorption of white light by indigo carmine dye solution over time after dilution and treatment with HC/plasma; comparison of time evolutions and decay fits of BPA, DF, E2, and PPZ with SupCaviPlasma device (25 °C, 53 W); degradation efficiency and reaction-rate constant of RhB removal as functions of solution conductivity; conventional technologies and AOPs for wastewater treatment; the properties, pros, and cons of various plasma processes; the schematic diagrams and relevant parameters of various HC/plasma; comparison of ^•^OH generation in various systems (PDF)

## Abbreviations

ACAP: acoustic cavitation-assisted plasma
AOPs: advanced oxidation processes
BPA: bisphenol A
CaviPlasma: alternating current discharge in a dense HC cloud in liquid
CBZ: carbamazepine
CIP: ciprofloxacin
DBD: dielectric barrier discharges
DC: doxycycline hyclate
DF: diclofenac
E2: 17β-estradiol
ED: electrical discharge
EY: energy yields
FUR: furosemide
GPD: glow plasma discharge
HC: hydrodynamic cavitation
HCAOP: HC-assisted O_2_ plasma process
HVCAP: high-voltage cold atmospheric plasma
MB: methylene blue
MO: methyl orange
MPAW-US: micronano bubbles plasma-activated water via ultrasonic stirring cavitation
MNZ: metronidazole
NOR: norfloxacin
NPX: naproxen
NTP: nonthermal plasma
OC: oxytetracycline dihydrate
PAW: plasma-activated water
PAW-DU: PAW with direct US
PAW-SU: pre-exposure of PAW and subsequent reactivation with US assistance
PD: plasma discharge
PPZ: propyphenazone
RhB: rhodamine B
ROS: reactive oxygen species
RONS: reactive oxygen and nitrogen species
SFX: sulfamethoxazole
sono-plasma: plasma treatment in a flow of cavitating liquid stream
STZ: sulfathiazole
SupCaviPlasma: HC combined with subatmospheric-pressure plasma
TC: tetracycline
TMD: tramadol
TMP: trimethoprim
US: ultrasound
UC: ultrasonic cavitation
UVC: ultraviolet C
VAL: valsartan
