# Supplementary material for: Combined Cavitation and Plasma in Water and Wastewater Remediation—A Review
Source: ACS Omega. 2026 Jan 8;11(2):2384–406. doi: 10.1021/acsomega.5c08760 (PMC12824758; doi:10.1021/acsomega.5c08760)
Supplement: Supplementary file 1 [file ao5c08760_si_001.pdf]

Combined cavitation and plasma in water and wastewater remediation - A review

Pengyun Liu[a], Subramaniam Chidambaranathapillai[a], Zhilin Wu[b,c*], Giancarlo Cravotto[a,*]

[a] Department of Drug Science and Technology, University of Turin, via P. Giuria 9, 10125 Turin, Italy.

[b] College of Chemistry and Chemical Engineering, Key (Guangdong-Hong Kong Joint) Laboratory for Preparation and Application of Ordered Structural Materials of Guangdong Province, Shantou University, Shantou 515063, China.

[c] Chemistry and Chemical Engineering Guangdong Laboratory, Shantou 515041, China

[*]Email: giancarlo.cravotto@unito.it, zhlwu@stu.edu.cn.

Supporting Information

Figures

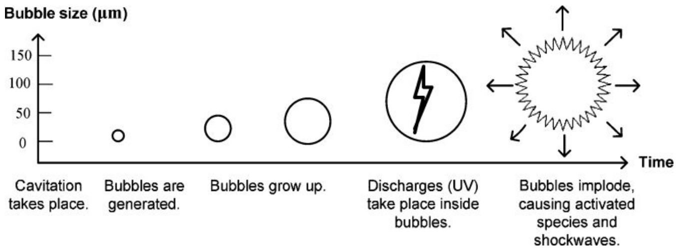

Figure S1. Possible mechanism of synergistic effect with US-assisted plasma treatment in microorganism inactivation. Reproduced from [100]. Copyright [2009] American Chemical Society.

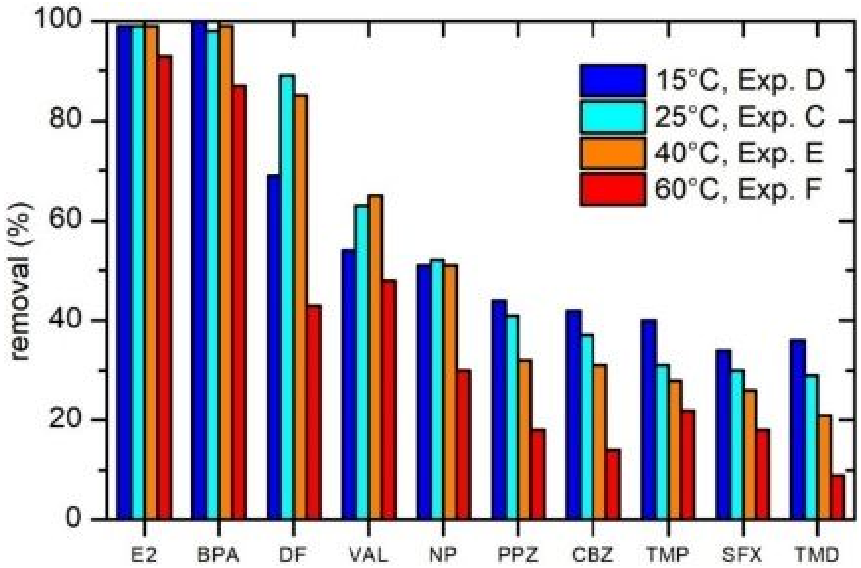

Figure S2. Removal of investigated micropollutants (100 μg/L of each) after 30 min of SupCaviPlasma treatment at different sample temperatures. Reprinted from ref. [101] Copyright (2024), with permission from Elsevier.

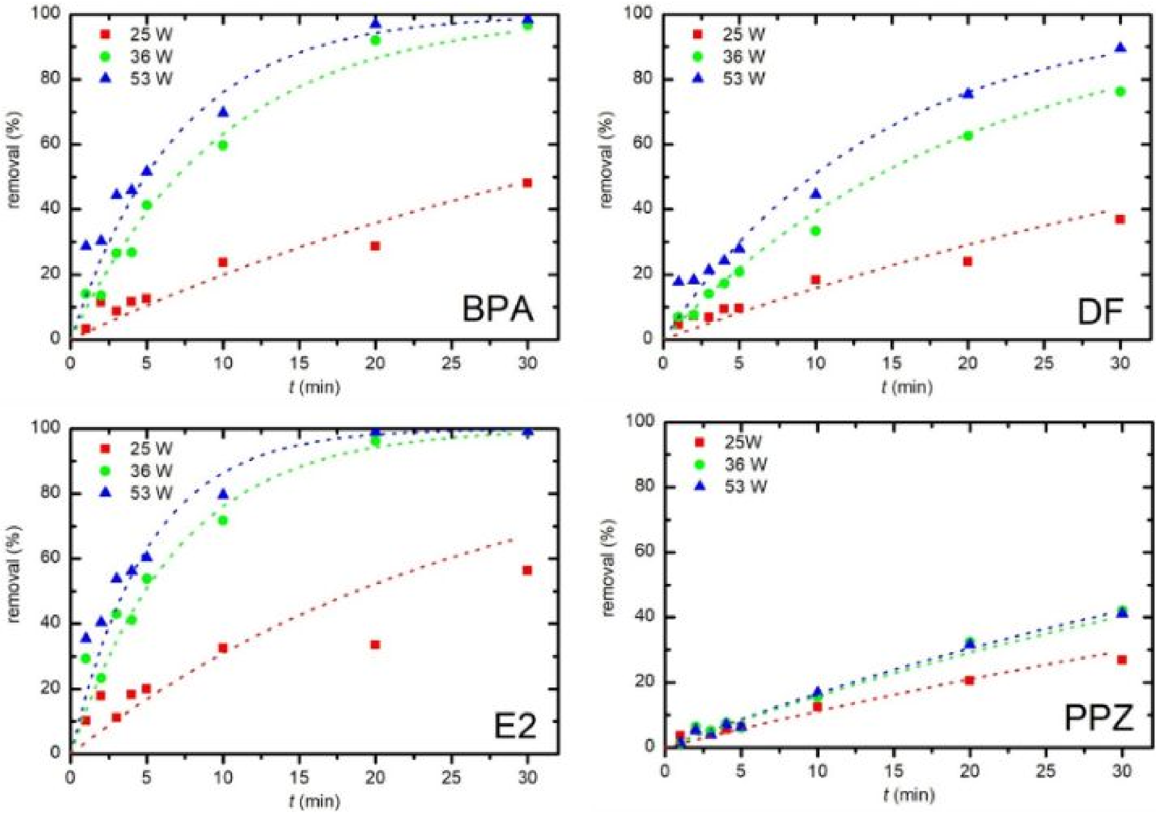

FigureS3. Removal of BPA, DF, E2, and PPZ during plasma-cavitation treatment (25°C) at different plasma powers. Reprinted from ref. [101] Copyright (2024), with permission from Elsevier.

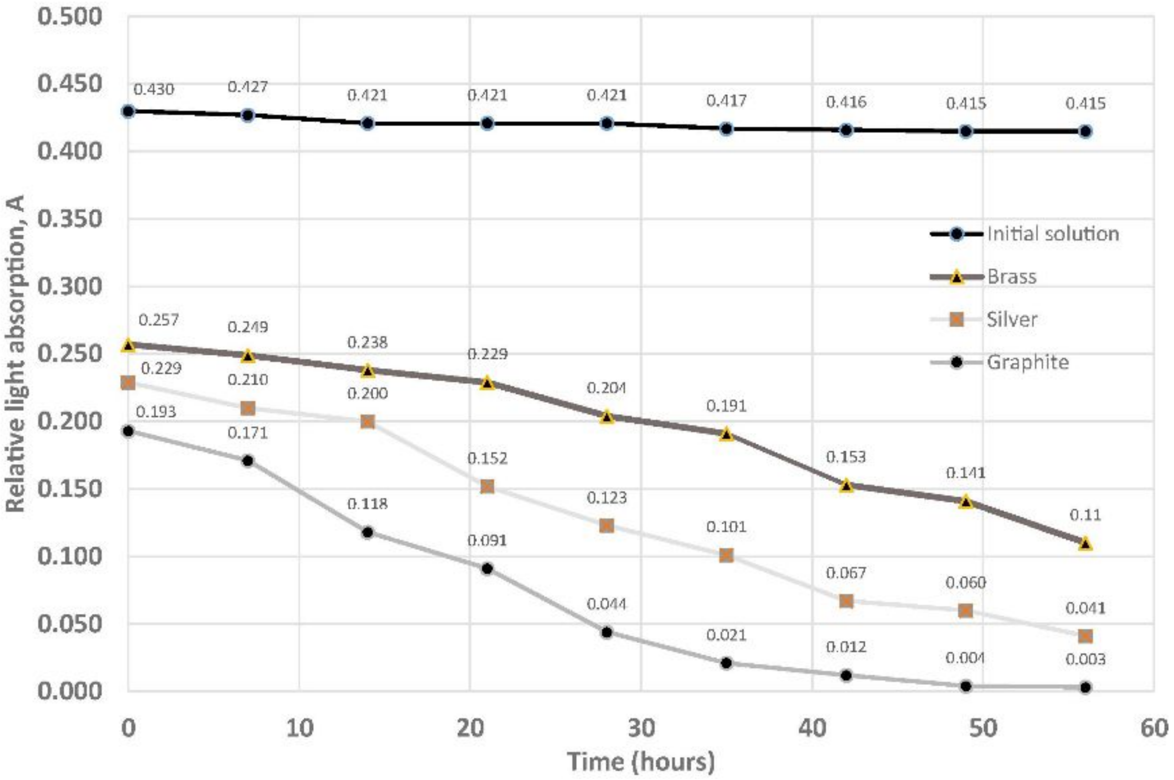

Figure S4. Change in absorption of white light by indigo carmine dye solution over time after dilution and treatment with HC/plasma. Reprinted from ref. [94] Copyright (2021), with permission from Elsevier.

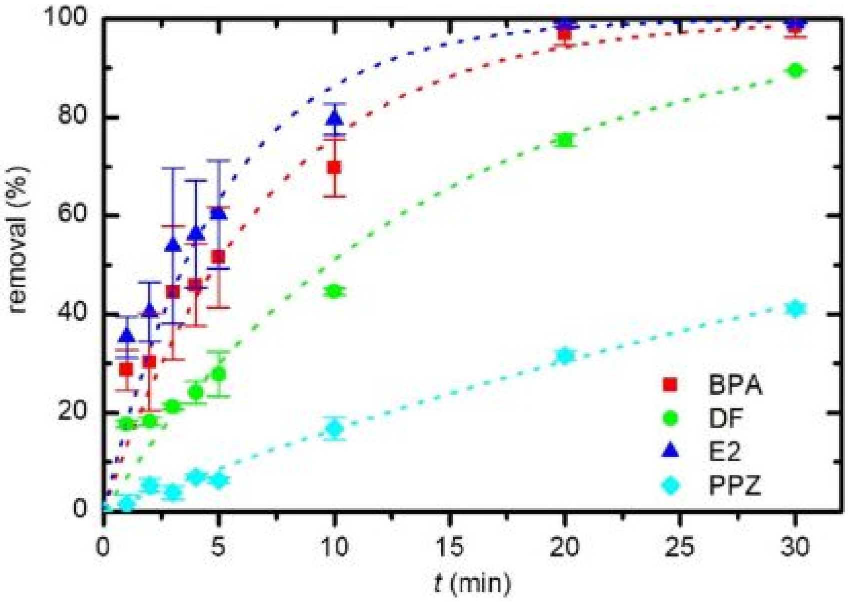

Figure S5. Comparison of time evolutions and decay fits of BPA, DF, E2, and PPZ with SupCaviPlasma device (25°C, 53 W). Reprinted from ref. [101] Copyright (2024), with permission from Elsevier.

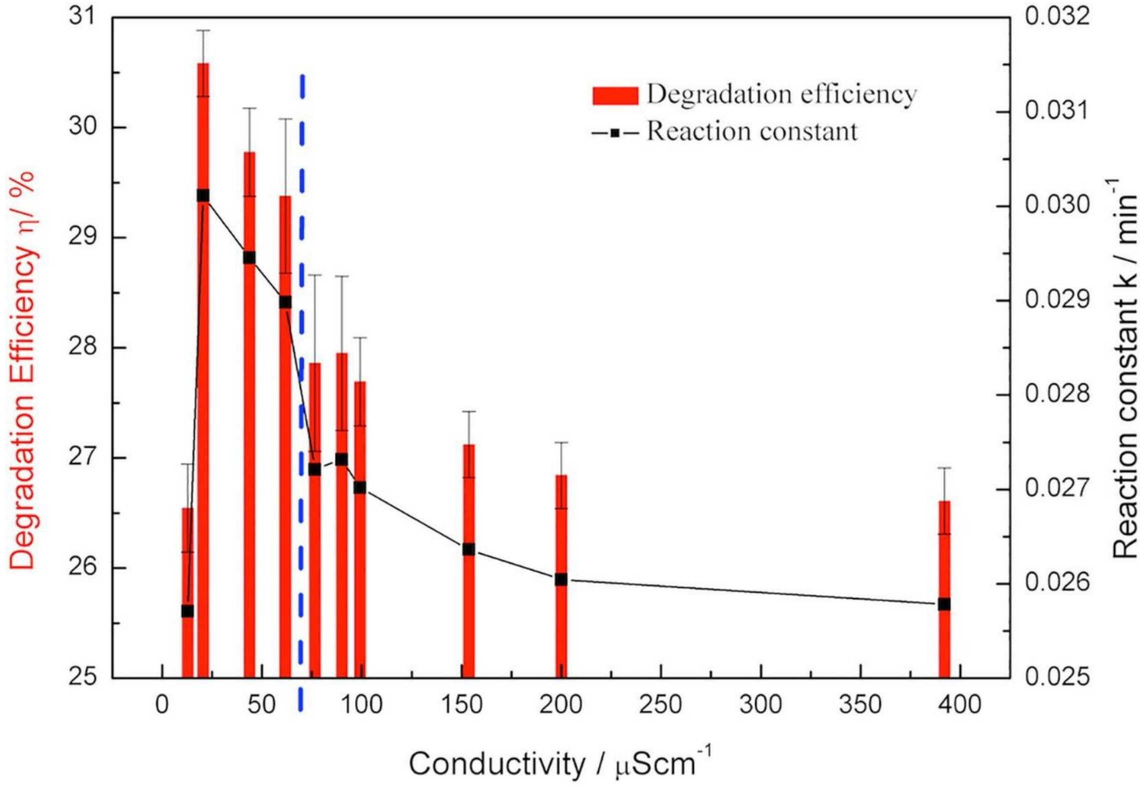

Figure S6. Degradation efficiency and reaction-rate constant of RhB removal as functions of solution conductivity. Reprinted from ref. [102] Copyright (2019), with permission from Elsevier.

Tables

Table S1 Conventional technologies and AOPs for wastewater treatment.

Contaminants	Processes	Pros	Cons	*Refs.*
Non-biotic contaminants	Chemical processes (*e.g.*, precipitation and flocculation)	Able to remove phosphorus; Microorganism residues after multi-stage aeration and separation can be suppressed by chemicals.	Poorly effective at low wastewater concentration; Standard discharges cannot be reached; possible to form organochlorine compounds (*e.g.*, chloroform, dichlorobromomethane, and chlorobromomethane, tribromomethane) and refractory compounds with metal ions (iron, zinc, manganese, cobalt, lead, copper and cadmium); Pathogenic microorganisms can develop resistance since constant interaction with chlorine and form antiseptic strains.	103–106
	Physicochemical processes (*e.g.*, adsorption, membrane filtration, coagulation, plasma, ultrasound (US), microwaves, and ultraviolet (UV) irradiation)	Free of significant reduction in use of chemicals; low cost; environmentally friendly; able to treat refractory organics and minimize by-products; enhanced by combination with AOPs (*e.g.*, photo- and sono-catalysis, Fenton-based processes, ozonation).	Physicochemical methods are often not thorough enough to treat pollutants, and pose secondary pollution and hidden risks. Adsorption only transfers the pollutant from water to another solid or liquid waste (non-destructive). Further treatments are required in membrane filtration and coagulation.	51,94,103,106–108
	Biological processes (*e.g.*, biofilm processes)	Activated sludge treated ~99% of wastewater in Germany; environmental-friendly; sustainable; able to treat large volumes of wastewater	Poor adaptability and versatility of microorganisms. Significantly decrease the activities of microorganisms, even fully inactivation.	103,104,106
	AOPs (*e.g.*, Fenton oxidation, electrochemical processes, photocatalysis, cavitation, plasmas, UV photolysis, wet oxidation, and so forth)	High reaction rates; reagent-free treatment methods usually; cost-effective; produces abundant reactive species, *e.g.*, hydroxyl radical ($^{\bullet}OH$), superoxide anion radical ($O_2^{\bullet-}$), single oxygen ($^1O_2$), H_2O_2, in water. Virtually no production of dangerous by-products. Each of these can be used alone or in a hybrid.	Strict operating conditions. The action of $^{\bullet}OH$ is highly limited by mass transfer. No individual process can cover everything; without secondary pollution, simplicity, economically feasible, environmentally friendly, robust, and easy to scale up.	101,102,105,108–110
	Fenton and Fenton-like processes	Fenton reagents (Fe^{2+}/H_2O_2) are powerful oxidants. Effective in degrading organic pollutants; Involves $^{\bullet}OH$ formation via H_2O_2 reaction with catalysts (*e.g.*, Fe^{2+})	Requires chemical reagents and that are difficult to recycle. Produces huge iron sludge and possible secondary pollution. Reactions are pH (2-3) dependent.	101,108,110,111
	UV photolysis	Promotes the formation of ROS, *e.g.*, $^{\bullet}OH$, from H_2O molecules by photolysis. Green technology.	High energy costs; water-quality dependent; High cost of equipment for UV treatment. Careful filtration is needed before UV irradiation.	101,109,110
	Ozonation	O_3 can directly react with organics and inorganics and self-decompose into powerful and unselective $^{\bullet}OH$, hydroperoxyl radical ($HO_2^{\bullet}$), and O_2. Able to remove organic contaminants and address taste and odor issues. Has a higher redox potential than O_2 and Cl_2. Mineralizes organics without producing toxic halogenated hydrocarbons.	Easily corrodes equipment; High energy costs. O_3 is a toxic gas. Risk of respiratory-system diseases; Poor dispersion or dissolution in water; persulfate and H_2O_2 may be needed; Causes assimilable organic carbon at high levels, leading to the bacterial contamination of treated water; Low effective for degrading resistant pollutants (*e.g.*, perfluorooctanoic acid).	94,99,107,111,112
	UV/O_3	High efficiency; Improved formation of reactive species; UV light can photolyze O_3, causing O_3 to form oxygen atoms in the excited and ground states, followed by rapid generation of H_2O_2 and $^{\bullet}OH$.	High energy consumption; high operation costs including ozone generation and device maintenance; risk of hazardous by-products and secondary pollution; high water quality (*e.g.*, turbidity and pH value) dependent; complex reactor design. The process was mass transfer controlled. Difficult to scale up; Requires combination with other methods or additional oxidation.	109
Bacterial contaminants	Filtration and sedimentation	Able to remove suspended and particulate matter; low production of hazardous by-products; reduces the use of disinfectants. Can be combined with other approaches; free of chemical residue; low energy consumption; and sustainable.	Insufficient inactivation of viruses; High costs; extensive infrastructure, frequent maintenance, and additional decontamination are required; Toxicity to aquatic organisms; Production of undesirable by-products.	96
	UV irradiation, chlorination, and heat treatment	Simple operation; Inactivates viruses by altering their native genome structure or capsid.	Insufficient inactivation of viruses; high costs; extensive infrastructure needed. Requires frequent maintenance and additional decontamination steps. Produces undesired and toxic by-products.	96
	Chlorination, ozonation, UV irradiation	Spectroscopic sterilization; prolonged oxidation; low cost of operation and equipment maintenance; efficient; able to	Uses toxic substances; expensive treatment; Chlorination is commonly harmful. Some microbes are resistant to chlorine.	94,113

		treat complex wastewater.		
Cyanobacterial blooms	Nutrient limitation; Semi-natural (*e.g.*, using plant extracts); and biological methods (*e.g.*, using probiotic microorganisms)	Low cost; low energy consumption; easy operation; free of or very low chemical usage; Sustainable.	Time-consuming; Water quality depends; Requiring to combine with other methods; Difficult to achieve complete purification.	[95]
	Physical process	Important for drinking-water treatment; Removes both cyanobacterial biomass and cyanotoxins.	Low efficiency; risk of secondary pollution; causes possible ecological disturbance; high costs; poor environmental adaptation; difficult to scale up; requires combination with other approaches.	[95]

Table S2 The properties, pros, and cons of various plasma processes.

Taxonomy	Category	Properties	Pros	Cons	*Refs.*
Distance of discharge plasma	Direct plasma in water (*e.g.*, pulsed arc)	i) Plasma is directly generated in the targeted water; ii) This process is in the evolvement stage.	i) Efficient reactive-species generation; ii) Strong electrical and thermal effects; iii) Free of additional chemical additives; iv) Simple and compact system design.	i) High breakdown voltage for plasma generation (up to 1 MV/cm for very pure water); ii) Increased electrical conductivity and difficulty in breaking down dissolved pollutants due to all the electrical energy being used for ion acceleration and then transformed to heat; iii) Lower plasma efficiency because of longer rise times of bubbles in liquid due to bubble generation preceding plasma ignition; iv) Impacted by power supply type, electrode material and geometry, and liquid properties; v) Possible second pollution by electrode wear; vi) Lower Energy Efficiency and Limited Penetration Depth.	[1,3,10,12,15,22,51,52,74]
	Direct plasma in water with the aid of gas or vapor bubbles (*e.g.*, Plasma Microbubbles)	i) Plasma is directly generated in the targeted water with the help of bubbles; ii) Higher temperatures (nearly the boiling point) are required to generate gas or vapor bubbles; iii) A needle and the water phase are used as electrodes.	i) The initiation and following discharge propagation facilitated by the bubbles and their large surface areas; ii) Higher energy yields (*EY*) than gas-liquid DBD; iii) Enhanced active species formation and plasma stability; v) Higher gas dissolution and mass transfer; vi) Slight electrode wear and improved plasma penetration.	i) Increased energy consumption; ii) Complexity in process control; iii) Possible formation of undesired by-products; iv) Higher equipment and maintenance costs; v) Possible effect of the existing gas.	[3,10,12,22,51,52]
	Indirect plasma (*e.g.*, electron beam)	i) Plasma is produced close to water surfaces (not directly inside liquids) by gas ionization and excitation (N_2, O_2, air); ii) Being highly developed, optimized, and commercialized; iii) Liquid (or electrode is immersed in liquid) is used as a counter electrode, and another electrode is put in the gas phase above the liquid.	i) Improved process control; ii) Uniform treatment over large areas; iii) Lower thermal impact; iv) Slight electrode wear and contamination; v) Scalable and able to be integrated with other methods.	i) Process efficiency is affected by liquid conductivities and gap distance; ii) The mass transfer of reactive species from gases into liquids may be restricted by low Henry's constants; iii) Lower plasma density; iv) Higher equipment costs; v) Possible secondary contamination.	[12,15,22,74]
	Remote plasma (*e.g.*, glow discharge, falling water	i) Plasma is ignited at one site in contact with water at a different site; ii) Being highly studied,	i) Uniform energy distribution; ii) Lower thermal impact;	i) Affected by electrode geometry, liquid properties, power supply types, *etc.*; ii) Less stable than direct plasmas;	[12,15,22]

	film)	improved, and commercialized;<br>iii) Plasma can be produced using electromagnetic or electric fields.	iii) Enhanced process control and improved uniformity;<br>iv) Scalable and flexibility;<br>v) Slight electrode wear and maintenance.	iii) Cost and equipment complexity;<br>iv) Challenge to control temperature;<br>v) Requiring high power to generate and maintain the plasma state;<br>vi) Uniform energy distribution and limited penetration depth;<br>vii) Possible safety and health risks.	
Phases of the PD	Gas-phase discharge plasma	i) Plasma is generated in gases injected above the water surface.	i) Easy to implement and efficient with significantly reduced discharge voltages;<br>ii) High plasma density and reactivity;<br>iii) Accurate process control;<br>iv) Uniform treatment over large areas;<br>v) Non-thermal, scalability and automation.	i) Short-lifespan active species with limited capability to penetrate the gas-liquid interface to react with contaminants in a restricted liquid depth;<br>ii) Causing water acidification due to the formation of undesirable chemical radicals in the gas phase and the subsequent dissolution in liquids;<br>iii) High energy consumption and complex equipment and maintenance;<br>iv) Possible secondary pollution, limited penetration depth, and complex process.	[12,15,19,35]
	Bubbling gas (to introduce bubbles near electrodes)	i) Plasma is produced in bubbles injected in liquids near electrodes;<br>ii) This process has been widely investigated in the last two decades.	i) Enhanced plasma reactivity and stability;<br>ii) Increased gas-liquid interaction;<br>iii) Slight electrode wear;<br>iv) Scalability for large-volume treatment;<br>v) Various gases can be used (O_2, Ar, N_2, and their mixture).	i) The reaction volume and pulse discharge are restricted by bubbling locations;<br>ii) Higher energy consumption;<br>iii) Possible adverse impact from bubbling and forming hazardous by-products;<br>iv) Limited penetration depth;<br>v) Complex process optimization.	[51,114]
	Use high voltage to water through its free surface (*i.e.*, DBD)	i) Plasma is produced at the free surface of water;<br>ii) This process has been widely tested in the last two decades.	i) More efficient than the bubbling-based methods in both technical and economic;<br>ii) Efficient for water treatment and disinfection with mild running conditions (non-thermal);<br>iii) Environmentally friendly and cost-effective;<br>iv) Localized plasma generation and efficient reactive species production;<br>v) Scalability and easy to hybrid with other systems.	i) Limited plasma density;<br>ii) Liquid quality dependence;<br>iii) Sensitive to water quality;<br>iv) Lower stability;<br>v) Poor uniformity control.	[23,51,114]
	Plasma-activated water (PAW)	i) Plasma is produced in gas and passes water through the plasma-activated system;<br>ii) Can be used for decontamination, pollutant degradation, biomass conversion, mitigating waterborne diseases, maintaining intact cell membranes in treated bacteria, *etc*.	i) Effective water treatment and disinfection;<br>ii) Environmentally friendly and non-thermal;<br>iii) Safety and sustainability;<br>iv) Free of additional chemical additives.	i) Limited plasma stability and short shelf-life;<br>ii) Limited treatment depth;<br>iii) Cost and equipment complexity;<br>iv) Possible side effects<br>v) Water quality dependence.	[3,46]
	Liquid-phase discharge plasma (*e.g.*, SupCaviPlasma, spark discharge, streamer (corona)	i) Plasma is directly produced inside water;<br>ii) This process can occur in spark and streamer mechanisms, highly affecting treatment performances;	i) Highly reactive species generation and uniform plasma-liquid interaction;<br>ii) Significantly depends on the composition of	i) Possible secondary pollution via electrode wear;<br>ii) High energy consumption and equipment costs;<br>iii) Limited penetration depth;<br>iv) Possible production of harmful by-products;	[12,15,19,35,58,84]

	discharge)	iii) Has been widely tested early by Clements *et al.* in 1987;<br>iv) The mechanisms include chemical reactions with highly active short-life radicals like •H, •OH, and •O and pyrolysis through high-temperature plasma channels (especially for volatile and hydrophobic contaminants);<br>v) The contaminants near or between electrodes favour their degradation.	wastewater;<br>iii) Free of external gas injection;<br>iv) Powerful electrical and thermal effects;<br>v) Versatile in various Liquid matrices;<br>vi) Localized and controllable plasma effects.	v) Complex process and optimization challenges;<br>vi) Requires very high breakdown voltage.	
	SupCaviPlasma	i) Plasma is produced only in vapor (water);<br>ii) The •OH is dominantly produced with less O_3, H_2O_2, and nitrates), and a low pH decrease.	i) Enhanced reactive species generation;<br>ii) Improved plasma stability and high energy efficiency;<br>iii) Stronger hydrodynamic and shockwave effects;<br>iv) Less electrode erosion and contamination;<br>v) Scalability for large-scale applications.	i) Complex system design and maintenance;<br>ii) Higher initial investment;<br>iii) Limited effectiveness sometimes;<br>iv) Risk of by-product formation;<br>v) Difficult process optimization.	[8]
	Pulsed spark discharge is directly used to water	i) Under constant conductivity and pH, the occurrence of the spark discharge is seen as a breakdown phenomenon.<br>ii) Needle-to-plate electrodes are commonly applied to break down water.	i) Generation of highly reactive species;<br>ii) Strong physical effects (shockwaves and cavitation);<br>iii) Free of additional chemical additives;<br>iv) Simple setup design;<br>v) Localized, very high-intensity energy delivery.	i) Requires very high voltages to initiate discharge, and high energy consumption;<br>ii) Low efficiency and very limited treatment areas;<br>iii) Possible secondary pollution via electrode wear and limited treatment volume per spark<br>iv) Potential for forming harmful by-products and adverse effects from bubbles.	[35,51,114]
	Streamer (corona) discharge	i) Only one electrode is usually required;<br>ii) Electrodes with wide surfaces yielding higher *REs*.	i) Non-thermal generation occurs at lower voltages;<br>ii) Generation of highly reactive species;<br>iii) Efficient gas-phase discharge and energy-efficient;<br>iv) Slight electrode wear;<br>v) Scalable and safe;<br>vi) With powerful plasma channels and UV emission.	i) Considerable energy is transformed into heat instead of degradation (particularly for refractory organic pollutants);<br>ii) Very challenging to form stable discharge plasma in liquids;<br>iii) Effective pollutant removal is mostly unachievable (especially at industrial levels);<br>iv) Localized plasma region and limited plasma penetration in liquids;<br>v) Relies on humidity and gas composition;<br>vi) Formation of unwanted by-products and risk of O_3 accumulation.	[51,114]
Gas and electron temperature	Direct thermal discharge plasma	i) Gas temperature up to 10,000 K for plasma generation.	i) Extremely high energy density and temperatures;<br>ii) Free of additional catalysts or additives;<br>iii) Complete decomposition of organics;<br>iv) High processing rates;<br>v) Operates at atmospheric pressure and is stable;<br>vi) Effective for hazardous waste removal.	i) Consumes less energy to create plasma environments;<br>ii) Severe thermal stress on equipment;<br>iii) High energy consumption and expensive setup;<br>iv) Not suitable for heat-sensitive applications;<br>v) Requires complex and intensive cooling;<br>vi) Generation of off-gas and by-products.	[15]

	Direct non-thermal (or cold) discharge plasma (NTP, *e.g.*, cold atmospheric plasma and low-pressure cold plasma)	i) Very high electron temperature (up to 10,000 K) compared to those of the ion and neutral (near room temperature);<br>ii) Mild gas conditions (room temperature and atmospheric pressure);<br>iii) Useful for water treatment, virus inactivation, destroying the cell walls of microalgae, and decontamination in medicine, textile manufacturing, *etc.*	i) Requiring less energy to create plasma environments with low energy consumption;<br>ii) Direct contact maximizes reactivity with high efficiency in removing emerging and recalcitrant water pollutants;<br>iii) Selective chemistry possible (targeted functionalization) and efficient for small-scale or surface-limited treatments;<br>iv) Minimal equipment stress and secondary pollution;<br>v) Green, fast, robust, flexible, portable, and scalable.	i) Limited penetration depth;<br>ii) Lower energy density;<br>iii) High energy expenditure, challenges in scalability, and expensive;<br>iv) Sensitive to environmental conditions;<br>v) Complex plasma-liquid interaction dynamics;<br>vi) Lower throughput at industrial levels.	[3,7,12–14,46,81,115]
	High-voltage cold atmospheric plasma (HVCAP)	i) For atmospheric pressure plasmas, sustaining them in noble gases is ideal to avoid massive loss of plasma radicals during collisions;<br>ii) This process has been extensively studied;<br>iii) Degradation via both interaction with UV and reacting with various RONS.	i) Operates at atmospheric pressure;<br>ii) Low temperature (safe for heat-sensitive targets);<br>iii) High chemical reactivity;<br>iv) Fast treatment with minimal equipment footprint;<br>v) Safe for consumers and environmentally friendly;<br>vi) Selective action due to its non-thermal nature.	i) Limited penetration depth and quite expensive when using noble gases;<br>ii) Requires careful insulation and shielding to prevent accidental exposure to high-voltage arcs or electromagnetic emissions;<br>iii) Risk of O_3 and NO accumulation;<br>iv) Needs specialized pulsed high-voltage power sources;<br>v) Sensitivity to operation conditions (*e.g.*, humidity, temperature, gas composition, and electrode configuration);<br>vi) Challenging uniform scale-up.	[8,13,81]
	Low-pressure cold plasma	i) Plasma is able to be easily maintained in any gas at low pressure.	i) Highly controlled conditions;<br>ii) Environmentally friendly and dry process;<br>iii) Low-temperature operation;<br>iv) High-purity atmosphere free of airborne contamination.	i) Requiring a vacuum system, huge space, and infrastructure;<br>ii) Batch processing, not continuous;<br>iii) Species cannot survive outside vacuums (plasma effects are limited inside the chamber).	[13]

Table S3 The schematic diagrams and relevant parameters of various HC/Plasma processes.

Order	Schematic diagrams	Systems	Capacity	HC conditions	Plasma conditions	*Refs.*
1	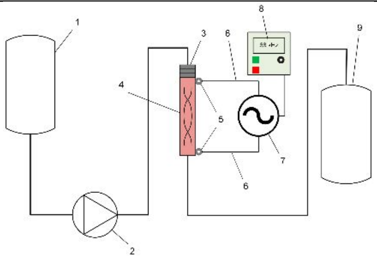<br>1-input water tank; 2-pump; 3-hydrodynamic emitter; 4-plasma reactor; 5-electrodes; 6-high-voltage cable; 7-generator; 8-frequency control; 9-output tank with treated water.	HC/Plasma	12 L/min at flow mode	Hydrodynamic emitter 40 bar	Alternating Voltage: 30-70 kHz and 4 kV	[110]

<table>
<tr><td></td><td></td><td></td><td></td><td></td><td></td><td></td></tr>
<tr><td>2</td><td>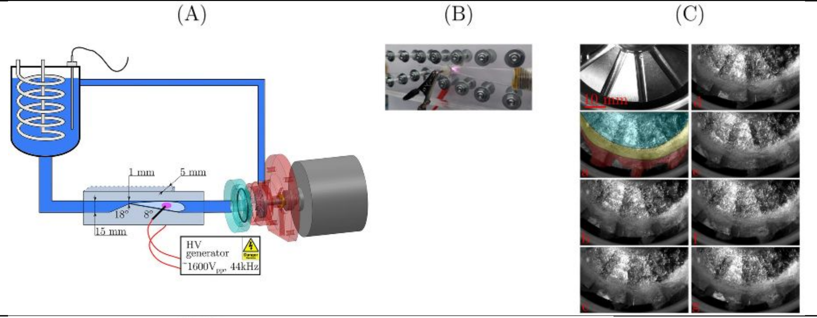
</td><td>Rotor-stator based SupCaviPlasma</td><td>5 L liquid circulated in a closed circuit</td><td>up to 250 bar, Venturi nozzle</td><td>Stainless-steel rod electrodes Direct voltage: 44 kHz and 1.63-1.68 kV</td><td>101</td></tr>
<tr><td>3</td><td>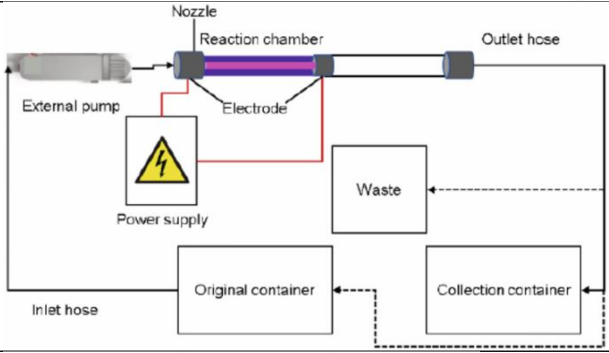
</td><td>HC/Plasma</td><td>20 L in batch mode and 2 L/min in flow mode</td><td>Hydrodynamic emitter up to 250 bar</td><td>Copper electrodes Alternating voltages: 30-50 kHz and 10-30 kV</td><td>104</td></tr>
<tr><td>4</td><td>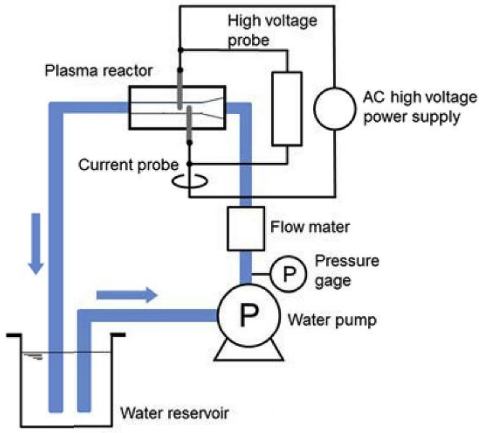
</td><td>HC/Plasma</td><td>2 L liquid circulated at 20 L/min</td><td>Nozzle</td><td>Alternating voltage: up to 1kV and 5-30 kHz</td><td>116</td></tr>
<tr><td>5</td><td>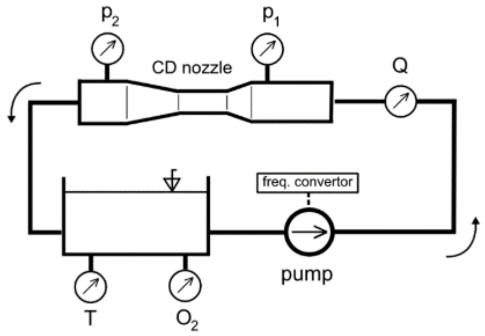
</td><td>HC/Plasma</td><td>6 L in continual flow-through mode</td><td>Converging-diverging nozzle 0-6 bar</td><td>PVC-insulated copper wire electrodes High-voltage:50 kHz and 400 W</td><td>95</td></tr>
</table>

6	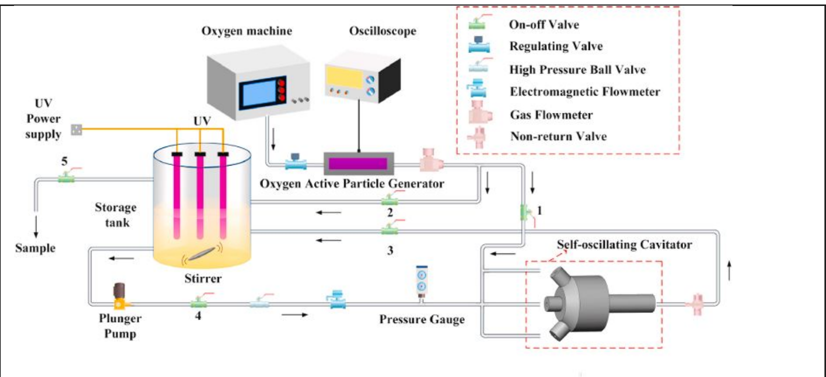	HC/DBD/UVC	5 L liquid circulated at 41 L/min	Self-excited reactor oscillation 12 bar	DBD: 46.11 W	109
7	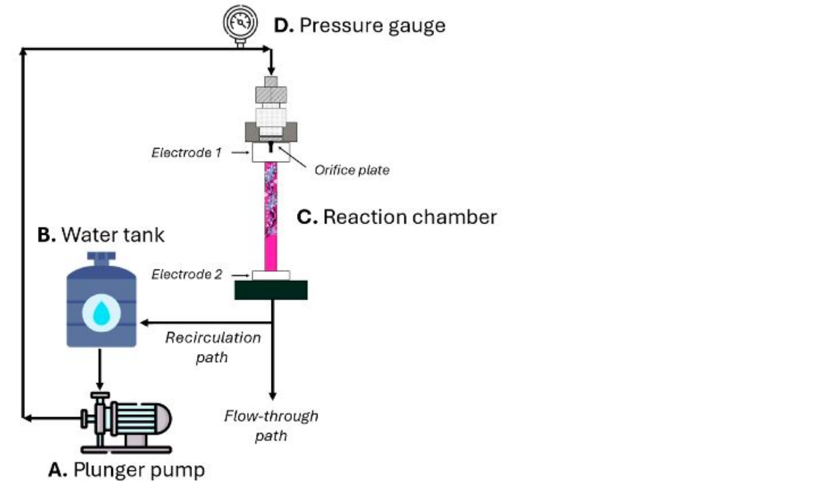	HC/ED plasma	15 L at a flow rate of 50 L/min	Rotor-stator reactor 10-20 bar	Alternating voltage: 15 kV and 48 kHz	107
8	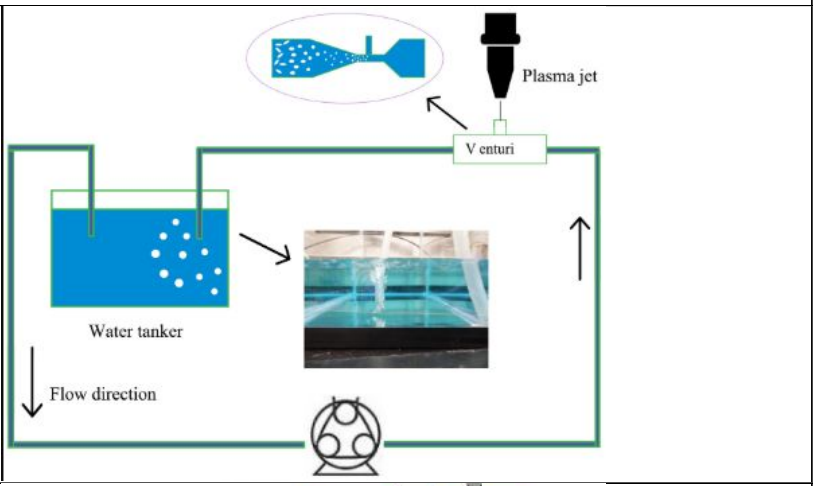	HC/Cold plasma	2-20 L at loop or flow mode (3-5 L/min)	venturi tube	Tungsten needle electrodes High voltage: 40 W and 15 kV)	106
9	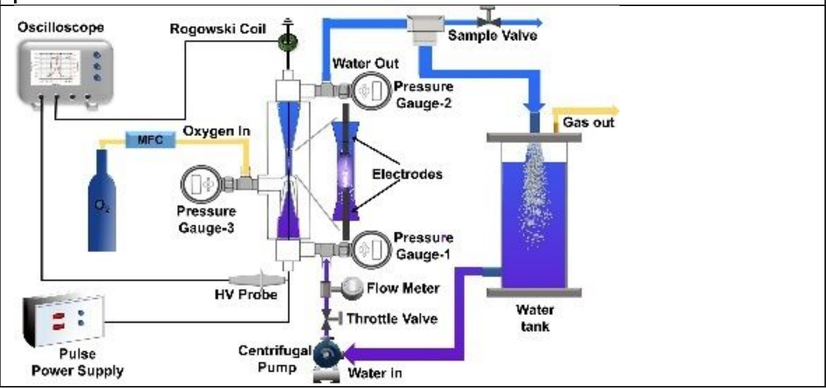	HCAOP	5 L at 3.1-9.5 L/min	Venturi's throat -5 to 5 bar	Tungsten needle electrodes. Solid-state nanosecond pulsed power: 50 kV and 1-1000 HZ	112

10	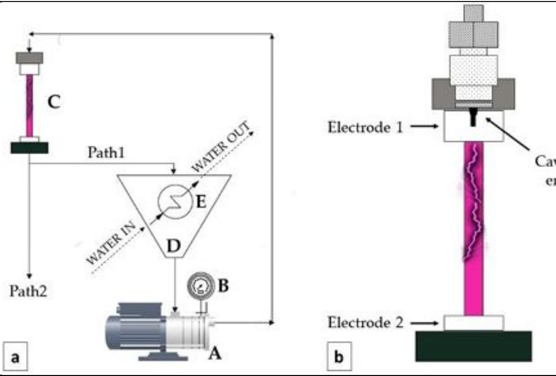	HC/Plasma	5 L at loop or flow mode	4-holed orifice plate 40-70 bar	Brass electrodes. Alternating voltage: 15 kV, 10-48 kHz, and 0-5 kW	111
11	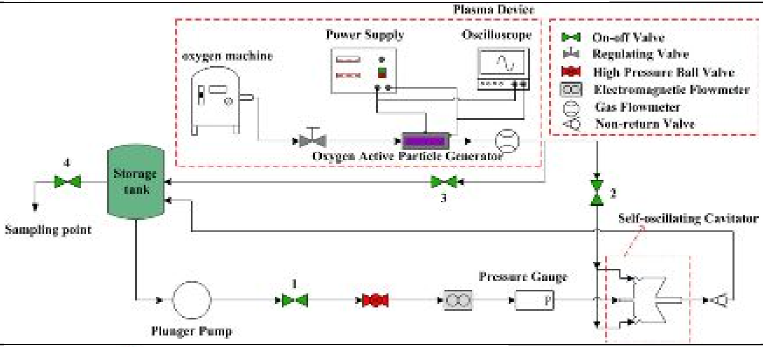	HC/Plasma	5 L	Self-excited oscillation 5-30 bar	-	103
12	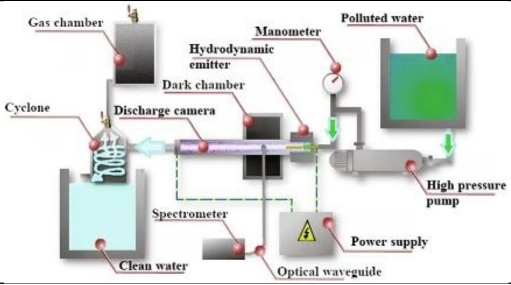	HC/plasma	16.7 L/min at flow mode	Hydrodynamic emitter 60 bar	Graphite, silver, and brass electrodes High voltage: 15 kV	94
13	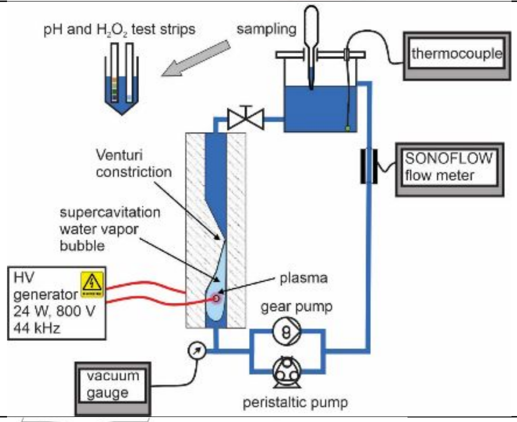	Super-cavitation/Cold plasm	0.43 L of recirculating water	Venturi throat 0.04-0.07 bar	Titanium rod electrodes. High voltage: 0.8 kV, 44 kHz, and 24 W	96
14	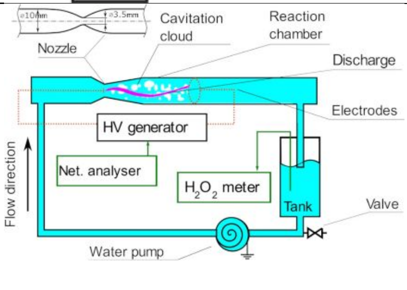	CaviPlasma	Circulating at 21.7 L/min at batch mode	Venturi nozzle	A titanium electrode and a ring-shaped steel electrode. Alternating high voltage: ~33 kHz, 10 kV, and 1 kW	97

15	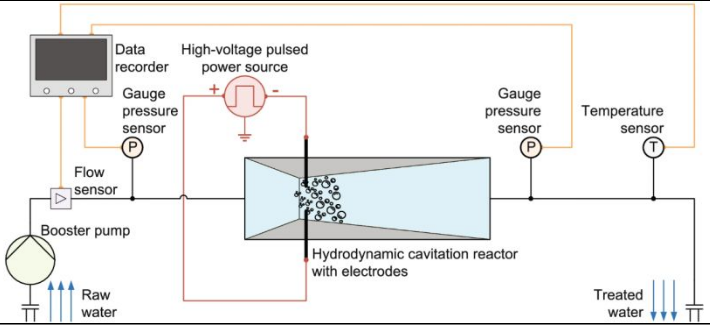	HC discharge plasma	15 L	-	-	[117]

Table S4 The schematic diagrams and relevant parameters of various UC/Plasma processes.

Order	Schematic diagrams	Systems	Capacity	UC conditions	Plasma conditions	*Refs.*
1	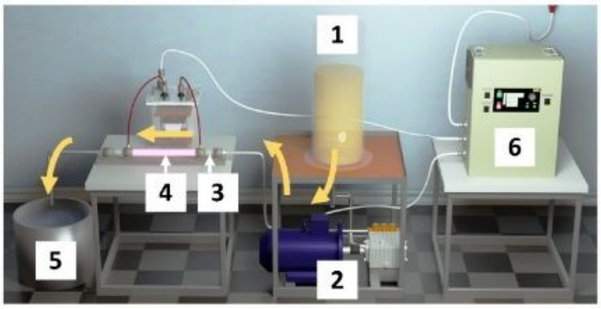 (1) contaminated water tank (CeO_2 catalyst is added to the water); (2) high-pressure pump; (3) hydrodynamic emitter; (4) discharge camera; (5) treated water tank; (6) power supply.	Sono-plasma	Continuous-flow at 16.7 L/min	Hydrodynamic emitter. Si_3N_4 and titanium cylindrical ultrasonic sonotrode. 0.3-60.0 kHz and 1.5–3.5 w/cm^2	Alternating voltage: up to 8.6 kV and 43 kHz	[99]
2	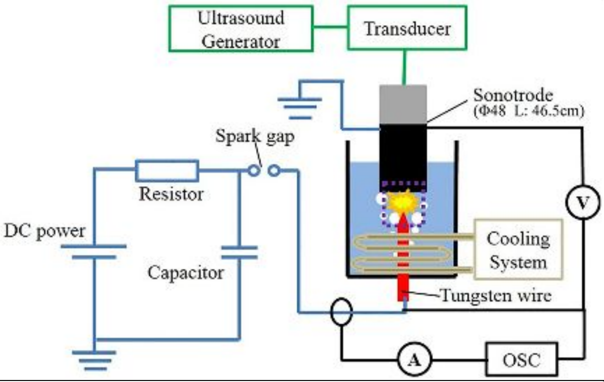	ACAP	Batch reactor	19-21 kHz and 210-300 W	Titanium and tungsten electrode. Pulse discharge voltage: 25-32 kV	[102,108]
3	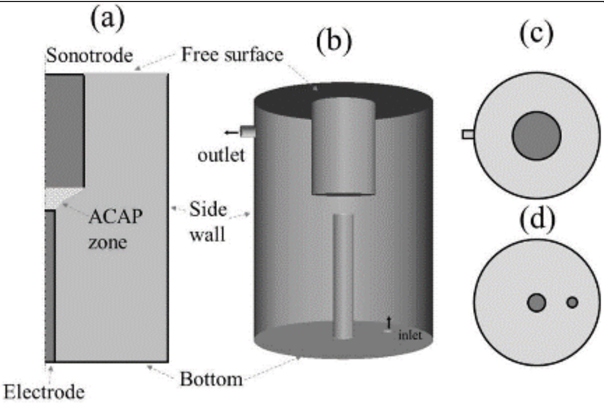 (a) ACAP zone location (b) arrangement of the main parts, (c) top view, and (d) bottom view	ACAP	2.5 L liquid was circulated at 2-8 L/min	19-21 kHz and 210-300 W	Titanium and tungsten electrode. Pulse discharge voltage: 25-32 kV	[51]

4	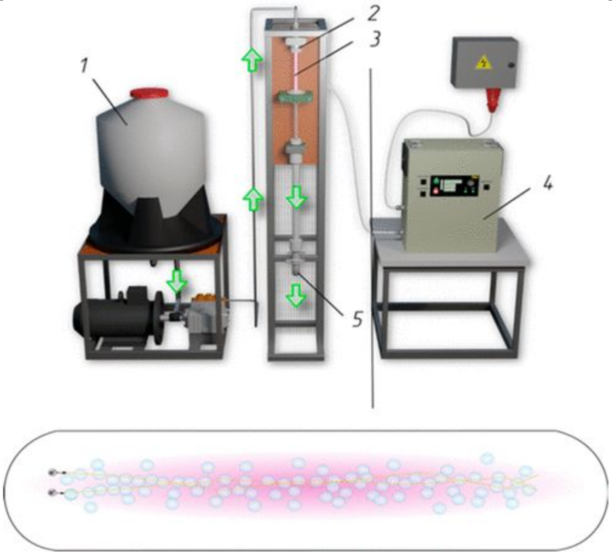<br>(1) feeding tank, (2) hydrodynamic emitter, (3) zone of cavitation inside the plasma reactor, (4) high-frequency generator of electric impulses, and (5) outlet.	Sono-plasma	-	Hydrodynamic emitter. 0.3-60 kHz 2.5–15.5 W/cm^2	Electric impulses. Alternating voltage: 25-38 kHz	[118]
5	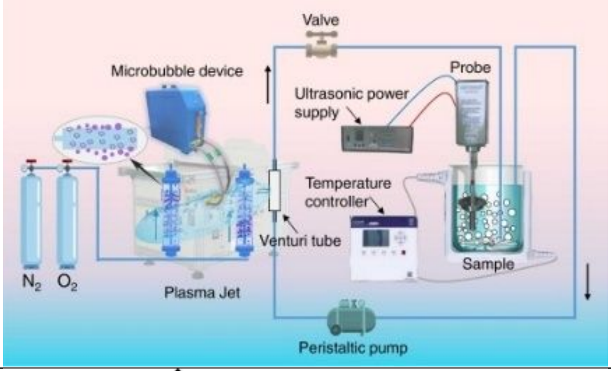	MPAW-US	10 L liquids were recirculated at 1 L/min	-	Voltage: 15 kV, 200-400 W	[113,119]
6	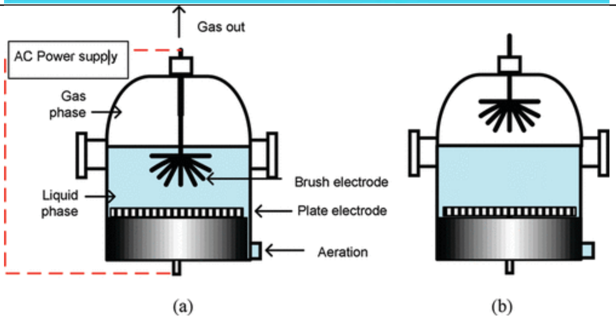<br>(a) the submerged reactor (b) the hybrid reactor	US-assisted plasma	-	140 W, 47 kHz	plate-type and brush-type stainless steel electrodes	[100]

Table S5 Comparison of $^{\bullet}$OH generation in various systems.

Processes	t (min)	*RE* without TBA (%)	*RE* with TBA (%)	*Refs.*
HC	30	9.54	2.20	[109]
UVC	30	17.91	11.76	
DBD	30	78.80	75.42	
HC/UVC	30	15.24	9.79	
UVC/DBD	10	66.45	58.54	
HC/DBD	10	83.03	76.66	
